# Supplementary material for: Narrowband room temperature phosphorescence of closed-loop molecules through the multiple resonance effect
Source: Nat Commun. 2024 May 28;15:4520. doi: 10.1038/s41467-024-48856-3 (PMC11133472; doi:10.1038/s41467-024-48856-3)
Supplement: Supplementary file 1 — Supplementary Information [file 41467_2024_48856_MOESM1_ESM.pdf]

Supplementary Information

Narrowband room temperature phosphorescence of closed-loop molecules through the multiple resonance effect

Xiaokang Yao[1,2,4], Yuxin Li[1,4] Huifang Shi[1,4], Ze Yu[1], Beishen Wu[1], Zixing Zhou[2], Chifeng Zhou[1], Xifang Zheng[1], Mengting Tang[1], Xiao Wang[2], Huili Ma[1], Zhengong Meng[1], Wei Huang[1,2,3] & Zhongfu An[1,2*]

[1] Key Laboratory of Flexible Electronics (KLoFE) & Institute of Advanced Materials (IAM), School of Flexible Electronics (Future Technologies), Nanjing Tech University (NanjingTech), Nanjing, China
[2] The Institute of Flexible Electronics (IFE, Future Technologies), Xiamen University, Xiamen 361005 Fujian, China.
[3] Frontiers Science Center for Flexible Electronics (FSCFE), MIIT Key Laboratory of Flexible Electronics (KLoFE), Northwestern Polytechnical University, Xi'an 710072, China.
[4] These authors contributed equally to this work.
** E-mail: iamzfan@njtech.edu.cn*

Contents

Suppl. Note 1. Statistical FWHMs of the RTP spectra based on the reported RTP molecules 2

Suppl. Note 2. Understanding the relationship between reorganization energy and the vibrationally-resolved electronic spectra 20

Suppl. Note 3. General procedures for the synthesis of phosphorescence emitters 22

Suppl. Note 4. Photophysical properties of organic emitters in solid and solution 33

Suppl. Discussion 1. Analysis of the host-guest energy levels 39

Suppl. Discussion 2. Energy transfer between the host and the guest component 41

Suppl. Note 5. Photophysical properties of the extended emitters 43

Suppl. Note 6. Supporting date for the application of narrowband RTP 47

Supplementary Reference 48

Suppl. Note 1. Statistical FWHMs of the RTP spectra based on the reported RTP molecules

Supplementary Table 1 | FWHMs of the phosphorescence spectra in the reported room-temperature phosphorescence materials.

Molecular names	FWHMs	Ref.
G-doped PVA films	100 nm	1
TPEDB-PVA	70 nm	2
4BBI/DIB	85 nm	3
PAMCz	70 nm	4
Br6A	85 nm	5
Br6	85 nm	
Br6A/Br6	85 nm	
Br6A	75 nm	6
DEOPh	70 nm	7
DECzT	80 nm	
DPhCzT	80 nm	
CzDClT	80 nm	
DCzPhP	100 nm	
2PtzO-3C	75 nm	8
2PtzO-4C	125 nm	
2PtzO-5C	125 nm	
2PtzO-6C	125 nm	
2PtzO-7C	75 nm	
2PtzO-8C	75 nm	
2PtzO-9C	75 nm	
2PtzO-10C	75 nm	
BDBT	80 nm	9
FBDBT	100 nm	
ClBDBT	80 nm	
BrBDBT	100 nm	
CD-CA	100 nm	10
CS-CH_3O	60 nm	11
CS-CH_3	60 nm	
CS-H	75 nm	
CS-Br	80 nm	
CS-Cl	60 nm	
CS-F	60 nm	

TPO-I	95 nm	12
TPO-Br	85 nm	
TPO-Cl	100 nm	
TPO-F	80 nm	
TPO-P	65 nm	
CZ-DBF	80 nm	13
CZ-DBFBr	70 nm	
CZ-DBT	80 nm	
CZ-DBTBr	60 nm	
PSSNa	120 nm	14
PSSLi	120 nm	
PSSK	120 nm	
PSSRb	120 nm	
$PSSNH_4$	120 nm	
NA/PCP	100 nm	15
NA/DCB	130 nm	
PDNA	100 nm	16
PDBA	100 nm	
rac-BINAP	120 nm	17
R-BINAP	110 nm	
S-BINAP	110 nm	
TCz-F-Cm	80 nm	18
TCz-F-Lab	55 nm	
TCz-H-Cm	140 nm	
TCz-H-Lab	90 nm	
CB[8]/BrBP–NH_2	80 nm	19
CT5-0	80 nm	20
CT5-20	80 nm	
TPA1	110 nm	21
TPA2	140 nm	
TPA3	170 nm	
TPA4	140 nm	
TPA5	150 nm	
SDP	90 nm	22
PzPh	80 nm	23
PzPh&BP	100 nm	
PzPh&TPO	90 nm	
PzPh&TPSi	90 nm	
PzPh&TPh	90 nm	
PzPh&SF	90 nm	
PzPh&TPA	85 nm	
TPM	100 nm	24
TPM-Cl	100 nm	

Py/BPO	80 nm	25
MOPy/BPO	85 nm	
MAPy/BPO	120 nm	
DMOPy/BPO	100 nm	
DMAPy/BPO	150 nm	
DPP-BOH-PVA	75 nm	26
(R, R)-DAACH	100 nm	27
(S, S)-DAACH	100 nm	
Cell-ImCNCl	115 nm	28
SI	120 nm	29
DBSI,	160 nm	
DBMI	170 nm	
DIMI	180 nm	
o-Br-TRZ	100 nm	30
m-Br-TRZ	100 nm	
p-Br-TRZ	95 nm	
CzA	70 nm	31
FA	80 nm	
PXZA	70 nm	
NTIA	100 nm	
OL	140 nm	32
DCz4Cl	70 nm	33
DCz4Br	70 nm	
DCz4I	70 nm	
ClCOF	80 nm	34
BrCOF	80 nm	
TSP	70 nm	35
DSP	70 nm	
TPP,	70 nm	
HSM	70 nm	
HPM	70 nm	
TNP	70 nm	
TMOT	80 nm	36
DMOT	80 nm	
CYAD	100 nm	
o-ITC	60 nm	37
m-ITC	65 nm	
p-ITC	65 nm	
2-diethylamino-9*H*-fluorene	60 nm	38
1CA	90 nm	39

Zn-TPA	70 nm	40
Zn-IPA	180 nm	
poly-BrBA	85 nm	41
poly-BrNp	85 nm	
poly-BrNpA	80 nm	
DBQPXZ	75 nm	42
24FPB	100 nm	43
SOBF-H	120 nm	44
SOBF-OMe	70 nm	
Ben-2S-C	100 nm	45
CF3-H-C	100 nm	
xerogel	120 nm	46
P1D1	70 nm	47
o-CzPh	80 nm	48
m-CzPh	80 nm	
p-CzPh	80 nm	
PVP–BrNpA	90 nm	49
PVP–BrHexene	130 nm	
PVP–BA	80 nm	
PNVCL–BrNpA	110 nm	
PNVCL–BrHexene	100 nm	
pSiTrz	100 nm	50
mSiTrz	110 nm	
CBTP-Cl	100 nm	51
CBTP-Br	100 nm	
CBTP-I	100 nm	
TX-OCH_3	110 nm	52
TX-OCH_2CH_3	110 nm	
TX-$OCH_2CH_2CH_3$	110 nm	
TX-$OCH(CH_3)_2$	120 nm	
PBA-IPA1	130 nm	53
PBA-IPA2	110 nm	
PMMA:TPB	110 nm	54
PMMA:TCATPB	105 nm	
TCATPB	90 nm	
Aba-*o*/-Cl	80 nm	55
Aba-*m*/-Cl	80 nm	
Aba-*p*/-Cl	100 nm	
Naba-*o*/-Cl	70 nm	
Naba-*m*/-Cl	70 nm	
Naba-*p*/-Cl	70 nm	

Npaba-*o*/-Cl	100 nm	
Npaba-*m*/-Cl	100 nm	
Npaba-*p*/-Cl	100 nm	
BP (doped into the epoxy polymer)	70 nm	56
BP (doped into the epoxy polymer)	100 nm	
TMPA (doped into the epoxy polymer)	90 nm	
TPA (doped into the epoxy polymer)	110 nm	
DPP (doped into the epoxy polymer)	130 nm	
PTCz	100 nm	57
PTF	150 nm	
SPh-TP	70 nm	58
WZ-12	90 nm	59
NpA-AM-Chol(I)	50 nm	60
NpA-AM-Chol(II)	50 nm	
NpA-Chol	50 nm	
BF_2-BP	70 nm	61
P-CDs	90 nm	62
P-CDs@B_2O_3	100 nm	
P-CDs@PVA	120 nm	
P-CDs@Urea	120 nm	
P-CDs@$Al_2(SO_4)_3$	120 nm	
P1	90 nm	63
P2	90 nm	
P3	90 nm	
P4	90 nm	
DB/CBBU	70 nm	64
CBBU	100 nm	
YAG	65 nm	65
WAG	60 nm	
LWAG	70 nm	
CF (DGEBA+DETA films)	100 nm	66
AM300	120 nm	67
AM303	130 nm	
AM315	120 nm	
AM323	130 nm	
AM115	120 nm	
AM515	120 nm	
OH-F-DPA-α-CD	70 nm	68
Poly-An-CD	90 nm	69
Poly-AC-CD	70 nm	

Ph-C8Br	100 nm	70
PhBr-C8	70 nm	
PhBr-C8Br	80 nm	
spiroBF_2-BP-1%	70 nm	71
spiroBF_2-MeBP-1%	70 nm	
spiroBF_2-EtOBP-1%	70 nm	
spiroBF_2-MeOBP-1%	70 nm	
CNS-455	80 nm	72
CNS-600	90 nm	
CNS-900	90 nm	
BPR (in pseudopolyrotaxanes)	70 nm	73
NPR (in pseudopolyrotaxanes)	80 nm	
c-CNT@BNO	100 nm	74
PA6/TPAB	80 nm	75
CTO-PVP	70 nm	76
CPO	70 nm	77
TMeOPPO	100 nm	
TMePPO	70 nm	
THPPO	120 nm	
TFPPO	100 nm	
TA (in PMMA)	120 nm	78
1TA1TA (in PMMA)	120 nm	
1TA2TA (in PMMA)	120 nm	
2TA2TA (in PMMA)	120 nm	
PMMA/LPM	80 nm	79
PMMA/LBC	150 nm	
NDI-PLA-100	80 nm	80
NDI-Br-PLA-100	80 nm	
NDI-Br-PCL-100	80 nm	
NDI-Ph-PLA-50	80 nm	
NDI-Ph-PLA-100	80 nm	
NDI-Ph-PLA-200	80 nm	
NDI-Ph-PLA-400	80 nm	
NDI- Br-Ph-PLA-50	80 nm	
NDI-Br-Ph-PLA-100	80 nm	
NDI- Br-Ph-PLA-200	80 nm	
NDI- Br-Ph-PLA-400	80 nm	
NDI-Br-Ph-PCL-100	80 nm	
NDI-Ph-PCL-200	80 nm	
NDI-Br-Ph-PLA-400	80 nm	
6C/CB[8]	90 nm	81
12C/CB[8]	90 nm	

12C/CB[8]/SCD	100 nm	
Ma&Ct	70 nm	82
Ma&Ae	110 nm	
Ma&Tm	70 nm	
Ma	70 nm	
PAA-24h-M1-0.1	100 nm	83
PAA-24h-M2-0.1	100 nm	
PAA-24h-M3-0.1	100 nm	
PAA-24h-M4-0.1	100 nm	
PAA-24h-M5-0.1	100 nm	
PAA-24h-M6-0.1	100 nm	
PAA-24h-M7-0.1	100 nm	
PAA-24h-M8-0.1	100 nm	
BLev (in boric acid matix)	100 nm	84
Cunano-clusters	110 nm	
DMAB	120 nm	85
p-DP	100 nm	86
p-DPSeZ	70 nm	
spiro-2N	80 nm	87
spiro-2C	120 nm	
BrPmDI	100 nm	88
IPmDI	100 nm	
Boric acid	110 nm	89
Czs-ph-3F	70 nm	90
CA	90 nm	91
BDA	70 nm	
CB5-0	90 nm	
MONI@BrBI	120 nm	92
MSNI@BrBI	120 nm	
MNNI@BrBI	100 nm	
RMM	100 nm	93
BMM	120 nm	
GMM	100 nm	
BPph-G	100 nm	94
dDBPph-G	80 nm	
Pph-G	180 nm	
NpA@PAC	100 nm	95
S-BN@PAC	120 nm	
HCM@PAC	120 nm	
QN@PAC	110 nm	
RhB@PAC	100 nm	

PF@PAC	150 nm	
o-BA,<br>m-BA<br>p-BA	100 nm<br>80 nm<br>80 nm	96
SFPz,<br>SF2Pz<br>SFPP	150 nm<br>150 nm<br>150 nm	97
G⊂CB[7]<br>G⊂CB[7]@SC4AD:RhB<br>G⊂CB[7]@SC4AD:DBT	100 nm<br>60 nm<br>150 nm	98
o-BFT<br>o-BFT@sPS	200 nm<br>110 nm	99
P-CN/CB[7]<br>P-CO_2Et/CB[7]<br>P-Me/CB[7]<br>P-CF_3/CB[7]	100 nm<br>80 nm<br>80 nm<br>80 nm	100
(RR)-BrPmDI<br>(SS)-BrPmDI<br>(RR)-Br_2PmDI<br>(SS)-Br_2PmDI	80 nm<br>80 nm<br>90 nm<br>90 nm	101
CBZCN/TPA<br>CBZCN/TPABr<br>TPACN/TPA<br>TPACN/TPABr	120 nm<br>120 nm<br>120 nm<br>120 nm	102
GA@PAA<br>GA@PAAM<br>GA@PVA<br>TA@SA<br>Cafa@SA<br>CA@SA	80 nm<br>100 nm<br>70 nm<br>70 nm<br>100 nm<br>120 nm	103
(R)-1	100 nm	104
1-BOX<br>3-BOX<br>5-BOX<br>7-BOX<br>9-BOX<br>11-BOX<br>13-BOX	70 nm<br>70 nm<br>70 nm<br>70 nm<br>70 nm<br>70 nm<br>70 nm	105
P2BA@BA<br>T2BA@BA	70 nm<br>90 nm	106
bTEoCN:CBP	120 nm	107

G⊂CB[8]@SC4AD/NiB	120 nm	108
CD-IQ/CB[7]	120 nm	109
spiroBF$_2$-PMMA<br>D-spiroBF$_2$-PMMA	80 nm<br>80 nm	110
DPCz-V1<br>TMPA-V1<br>HDCz-V1<br>PyB-V1	120 nm<br>120 nm<br>100 nm<br>120 nm	111
DPA (in KCl matrix)<br>TPP (in KCl matrix)<br>2MBP (in KCl matrix)	100 nm<br>180 nm<br>170 nm	112
G/CB[8]<br>G/CB[8]-Gel/EY	80 nm<br>150 nm	113
C24C8@PVA	100 nm	114
DDCzIPN	80 nm	115
TPA-DCPP	150 nm	116
Cz-BP<br>Cz-DPS<br>BCz-DPS<br>BCz-BP	100 nm<br>80 nm<br>80 nm<br>80 nm	117
CzPBr<br>CzPCl<br>CzBBr<br>CzBCl	90 nm<br>85 nm<br>85 nm<br>85 nm	118
PA<br>α-PA<br>β-PA<br>MA<br>α-MA<br>β-MA	100 nm<br>100 nm<br>130 nm<br>100 nm<br>110 nm<br>110 nm	119
XCO-Ph<br>XCO-PhCl<br>XCO-tBu<br>XCO-PiCl	100 nm<br>95 nm<br>95 nm<br>100 nm	120
TBP-CB[8]	90 nm	121
PVP-S<br>PVP-C<br>PVP-B	110 nm<br>110 nm<br>110 nm	122

BrFlu-CBr	80 nm	123
MCzT	75 nm	124
ECzT	70 nm	
PCzT	75 nm	
BCzT	75 nm	
FCzT	70 nm	
poly-Br-An	80 nm	125
poly-Br-An-MA	75 nm	
poly-Br-An-Cp	75 nm	
T3-*m*-PyCzBr	80 nm	126
T2-*m*-PyCzBr	80 nm	
PH/CB[7]	75 nm	127
PBr/CB[7]	90 nm	
FDA/TPP	70 nm	128
DTA/TPP	80 nm	
DBC/TPP	85 nm	
DQD/TPP	85 nm	
FDA/TPAS	75 nm	
DTA/TPAS	80 nm	
DBC/TPAS	85 nm	
DQD/TPAS	85 nm	
S-COOCz	75 nm	129
R-COOCz	75 nm	
COOCz	70 nm	
PCP-BrCz	85 nm	130
PPCP-BrCz	70 nm	
CBA	80 nm	131
CSA-I	100 nm	
PYI	120 nm	132
PYBr	110 nm	
PYCl	80 nm	
$PYPF_6$	120 nm	
o-MCBA	75 nm	133
m-MCBA	75 nm	
p-MCBA	85 nm	
4C	120 nm	134
Ph	120 nm	
MeOPh	120 nm	
MeO_3Ph	120 nm	
4C-Br	100 nm	
COOH-Br	100 nm	
PLA-Br	100 nm	
PLA-OPh-Br	100 nm	

Chi-Br	100 nm	
Br-BSA	100 nm	
PNA/DBBP	70 nm	135
Br-PNA/DBBP	70 nm	
PNA-Br/DBBP	130 nm	
$CzS-CH_3$	80 nm	136
$CzS-C_2H_5$	80 nm	
C1-crosslinked NPs	80 nm	137
AHT	100 nm	138
SF2C	70 nm	139
SFPC	100 nm	
SF2P	100 nm	
CTO@PVA	80 nm	140
TAT-PVA	80 nm	141
CTAT-PVA	70 nm	
CS-PVA	80 nm	
C4-Br	80 nm	142
C4-I	60 nm	
DPPZ	75 nm	143
PSS	90 nm	144
p-BrTCzv	80 nm	145
m-BrTCz	80 nm	
o-BrTCz	80 nm	
Br6A@PMMA	100 nm	146
$H-NPCzBF_2$	100 nm	147
$Br-NPCzBF_2$	100 nm	
$I-NPCzBF_2$	100 nm	
(R)-PNA	105 nm	148
(S)-PNA	110 nm	
(R)-PBNA	112 nm	
(S)-PBNA.	110 nm	
DPCz-doped PVA	90 nm	149
DBCz-doped PVA	66 nm	
Cz-doped PVA	75 nm	
DBF-doped PVA	133 nm	
DBT-doped PVA	75 nm	
AQPY⊂CB[8]_synthesized	90 nm	150
AQPY⊂CB[8]_photooxidized	88 nm	
ANPY⊂CB[8]	70 nm	

Foam-0.15 gmL^{-1}	110 nm	151
Foam-0.10 gmL^{-1}	120 nm	
Foam-0.07 gmL^{-1}	140 nm	
Foam-0.05 gmL^{-1}	140 nm	
Foam-0.03 gmL^{-1}	145 nm	
DECzP	150 nm	152
DECzPO	120 nm	
DECzPS	90 nm	
HCCP−HHTP−EA	75 nm	153
HCCP−HHTP−Q	75 nm	
HCCP−HHTP−THBP	75 nm	
HCCP−HHTP−SDP	75 nm	
HCCP−HHTP−BP	75 nm	
HCCP−HHTP−PCA	75 nm	
BrPmDI-LP	80 nm	154
1	90 nm	155
2	90 nm	
4	70 nm	
5	80 nm	
6	75 nm	
7	75 nm	
8	130 nm	
9	80 nm	
10	120 nm	
11	110 nm	
12	110 nm	
13	100 nm	
15	80 nm	
17	110 nm	
18	120 nm	
19	60 nm	
20	110 nm	
BrNp-β-CD	85 nm	156
BrHB-β-CD	90 nm	
BrBp-β-CD	100 nm	
BrNpA-β-CD	100 nm	
MA-IPA	100 nm	157
MA-TPA	90 nm	
MC/BP	140 nm	158
P1a2a3a/BP(1;100)	>200 nm	
P1a2a3a/BP(1;1000)	>200 nm	
P1a2a3a/BP(1;500)	190 nm	
P1a2a3a/BP(1;50)	200 nm	

P1a2a3a/BP(1;25)	120 nm	
DM-7HIT	150 nm	159
DM-7MIT	150 nm	
CDOFs	70 nm	160
HCB-PVA	65 nm	161
F1-PU without acid	100 nm	162
py@OA_2	80 nm	163
DMPBA	110 nm	164
MPBA	110 nm	
CPBA	115 nm	
BBA	120 nm	
APBA	90 nm	
FPBA	50 nm	
TCNPh	70 nm	165
*p*CNPh	100 nm	
*m*CNPh	100 nm	
*o*CNPh	100 nm	
1a	90 nm	166
1b	80 nm	
1c	90 nm	
PQ1 (doped in PMMA)	90 nm	167
PQ2 (doped in PMMA)	90 nm	
PQ3 (doped in PMMA)	100 nm	
1	120 nm	168
2	115 nm	
3	100 nm	
4	100 nm	
5	140 nm	
BPA 3-PVA	90 nm	169
BPNa 3-PVA	90 nm	
BPE 3-PVA	150 nm	
PBAM 3-PVA	100 nm	
ACE	87 nm	170
THF-IPR	87 nm	
CHL-IPR	87 nm	
THF-ETH	75 nm	
ACN	70 nm	
SiAz (in Zeonex)	125 nm	171

$ACAC^{-}/NH^{4+}$<br>$ACAC^{-}/Et_2NH^{2+}$<br>CF_3AC^{-}/Et_2NH^{2+}<br>CF_3AC^{-}/NH^{4+}	125 nm<br>100 nm<br>110 nm<br>110 nm	172
CEimCl<br>CMimCl<br>CiPimCl<br>CPimCl<br>CBimCl<br>COHimCl<br>CBeimCl<br>CMimCl<br>CC4MimCl<br>CC5MimCl	100 nm<br>125 nm<br>150 nm<br>75 nm<br>75 nm<br>125 nm<br>90 nm<br>125 nm<br>100 nm<br>83 nm	173
m,p/CDs-ME<br>d,p/CDs-ME<br>e,p/CDs-ME<br>o,p/CDs-ME	125 nm<br>125 nm<br>100 nm<br>130 nm	174
NapBr@Pg<br>PyBr@Pg	100 nm<br>75 nm	175
SDM1 film	175 nm	176
BDA@Lap	135 nm	177
D1 (β-estradiol)<br>A3 (β -estradiol)	120 nm<br>110 nm	178
Br6A	99 nm	179
BzylPSeZ	100 nm	180
L2Q6X	100 nm	181
BTPO	98 nm	182
TRZ-PHT	102 nm	183
PAM/SA	66 nm	184
BrPCN-r	97 nm	185
Nacpb	150 nm	186
CZBA	80 nm	187
CDs-PVA	110 nm	188

DCB	102 nm	189
Poly-NpA-SeO	95 nm	190
IDCA	110 nm	191
CDs	125 nm	192
TMeOPPO	98 nm	193
CPCN	81 nm	194
M-CA	105 nm	195
MBA	80 nm	196
CzS-*o*-py	90 nm	197
DAC	110 nm	198
TBCzSP	125 nm	199
Chr-Ph-3-Cz	80 nm	200
TPA-FL-CN	75 nm	201
CEL@PAA	125 nm	202
N-CDs/MA	124 nm	203
CzIP	122 nm	204
PthBr	102 nm	205
CNQDs-CMC	130 nm	206
AqC6	120 nm	207
BNDs@B_2O_3	115 nm	208
PMCl/phCB[6]	90 nm	209
P1	80 nm	210
CzC8Cz	100 nm	211
DCzCs-Ac	95 nm	212

CZBP	70 nm	213
BCZBP	100 nm	
DBCZBP	70 nm	
BrBNT 1/BrBDBT	70 nm	214
BrBNT 2/BrBDBT	80 nm	
BrBNT 3/BrBDBT	100 nm	
$BrNpA_2DMA_{79}$*m*$TEGA_{19}$/NHT	100 nm	215
TPB (in PMMA)	100 nm	216
BipyDpa (in PMMA)	100 nm	
BipyTpa (in PMMA)	100 nm	
PhenDpa (in PMMA)	70 nm	
PhenTpa (in PMMA)	100 nm	
DCED	100 nm	217
o-PBCM	100 nm	
m-PBCM	100 nm	
p-PBCM	100 nm	
CPM	120 nm	218
CMPM	60 nm	
CMOPM	70 nm	
BDPDPPT (in Exceval)	70 nm	219
BDPB-Ac (in Exceval)	70 nm	
BDPB-OH (in Exceval)	100 nm	
BDPB (in Exceval)	100 nm	
PBHDB@PMMA film	90 nm	220
G⊂CB[7]@HACD	100 nm	221
SA-BP	110 nm	222
SA-TPE	120 nm	
SA-Py	90 nm	
s-DTBT	70 nm	223
d-DTBT	90 nm	
t-DTBT	100 nm	
$M–CH_3$	80 nm	224
BCZ1	110 nm	225
BCZ2	120 nm	
BCZ3	70 nm	
BCZ4	100 nm	
CzBdBr@BBP	90 nm	226
CzBdBr@DMAP	90 nm	
CzBdBr@TPA	130 nm	
DPANa@PMMA	90 nm	227

PhCz<br>CPhC<br>BPhCz	70 nm<br>70 nm<br>70 nm	228
MCATMA<br>MCAIPA<br>MCATPA<br>MCAPA	60 nm<br>60 nm<br>70 nm<br>90 nm	229
OSNs1<br>OSNs2<br>OSNs3	120 nm<br>130 nm<br>120 nm	230
1/CB[8]	150 nm	231
DPANa (in PMMA)	90 nm	232
1<br>2<br>3<br>4<br>5	90 nm<br>90 nm<br>100 nm<br>100 nm<br>70 nm	233
mTPA	60 nm	234
DNCzP<br>DNCzPO<br>DNCzPS	90 nm<br>100 nm<br>80 nm	235
Trzpy<br>DCF-10	120 nm<br>140 nm	236
H-PzPh<br>F-PzPh<br>CF_3-PzPh<br>OCH_3-PzPh	80 nm<br>80 nm<br>80 nm<br>80 nm	237
TPTZPO<br>PTZPS<br>TPTZPSe	90 nm<br>110 nm<br>100 nm	238
CDs/PVP film	90 nm	239
Br_1NTE<br>Br_2NTE<br>Br_4NTE	60 nm<br>80 nm<br>80 nm	240
0M<br>1M<br>2M	120 nm<br>70 nm<br>90 nm	241
PB-OH<br>PB-MeO<br>PB-EtO<br>PB-PrO	100 nm<br>100 nm<br>100 nm<br>90 nm	242

PB-BuO	90 nm

Suppl. Note 2. Understanding the relationship between reorganization energy and the vibrationally-resolved electronic spectra

The reorganization energy: The reorganization energies between ground state and excited state can be expanded into the reorganization energies of each vibrational mode,

$$\lambda = \sum \lambda = \sum \hbar\omega S \quad \text{(Equation 1)}$$

$$\lambda = \frac{k}{2}\Delta Q^2,\ S = \frac{\lambda}{\hbar\omega} \quad \text{(Equation 2)}$$

$$S = \frac{1}{2\hbar}\omega\Delta Q^2 \quad \text{(Equation 3)}$$

Here λ represents the total reorganization energy; *S* is the Huang-Rhys factors of each normal mode; k and ω are the corresponding force constants and vibrational frequencies.

It should be noted that, the normal coordinates of the ground state and the excited state obtained by projecting the coordinates along the vibrational mode Q_1 and Q_2 are different and follow a linear transformation relationship,

$$Q_1 = \mathrm{J} Q_2 + \Delta Q \quad \text{(Equation 4)}$$

Here J is Duschinsky matrix, and ΔQ is the displacement in the canonical coordinate.

Vibrationally-resolved electronic spectra: Under the condition of Born–Oppenheimer approximation and Franck–Condon approximation, the vibrationally-resolved electronic spectrum is obtained from overlap integrals $\mathrm{FCI}(m,n) = \langle \Phi_m(Q_1) | \Phi_n(Q_2) \rangle$ of the vibrational wave function (*Φ*, Q, m and n represent the wave function, nuclear displacement, initial and final vibrational quantum numbers of each vibrational modes), namely the Franck–Condon (FC) factor. If the Duschinsky matrix is ignored, i.e., J = *I*, the multidimensional FC factor is obtained an approximate formula in the literature[242]:

$$\mathrm{I}(\mathrm{m_1,n_1,m_2,n_2,\ldots,m_p,n_p}) = \prod_{\mathrm{i=1}}^{\mathrm{p}} \mathrm{FCI}(\mathrm{m_i,n_i})^2 \exp\left(\frac{-\hbar \mathrm{m_i}\omega_{\mathrm{i}}}{\mathrm{k_B T}}\right) \quad \text{(Equation 5)}$$

$$\mathrm{FCI(m,n)}^2 = \exp(-S) S^{\mathrm{n-m}} \frac{\mathrm{m!}}{\mathrm{n!}} \left[L_{\mathrm{m}}^{(\mathrm{n-m})}(S)\right]^2 \quad \text{(Equation 6)}$$

m and n are the initial and final vibrational quantum numbers of each vibrational modes, p is the p mode of vibration, k_B is the Boltzmann constant, *S* is Huang-Rhys factor, T is the temperature, and $L_m^{(n-m)}(S)$ is the Laguerre polynomial.

It is also pointed out in the literature that when only considering the transition from the lowest vibrational state of initial state to the final state, it can be further approximated,

$$\mathrm{I}(0,\mathrm{n_1},0,\mathrm{n_2},\ldots,0,\mathrm{n_p}) = \prod_{\mathrm{i=1}}^{\mathrm{p}} \mathrm{FCI}(0,\mathrm{n_i})^2 \quad \text{(Equation 7)}$$

$$\mathrm{FCI}(0,n)^2 = \frac{S^n}{n!}\exp(-S) \quad \text{(Equation 8)}$$

In the approximate equation, $\mathrm{FCI}(0,n)^2$ turn into the Poisson distribution, and the variance equals Huang-Rhys factor *S*. Therefore, the increase of the Huang-Rhys factor of each vibrational modes can lead to the increase of the full width at half maxima.

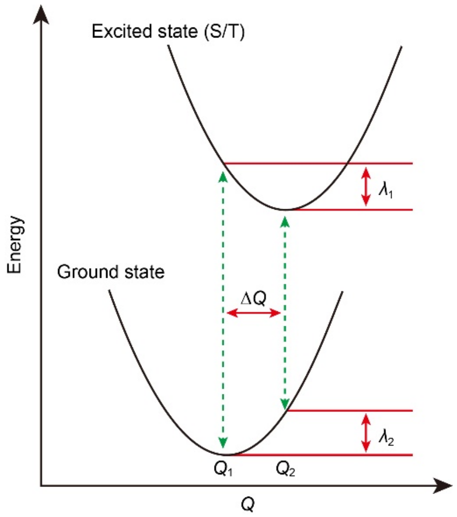

Supplementary Figure 1 | Sketch of the potential energy surfaces for the excited and ground states. Q represents nuclear displacement.

Suppl. Note 3. General procedures for the synthesis of phosphorescence emitters

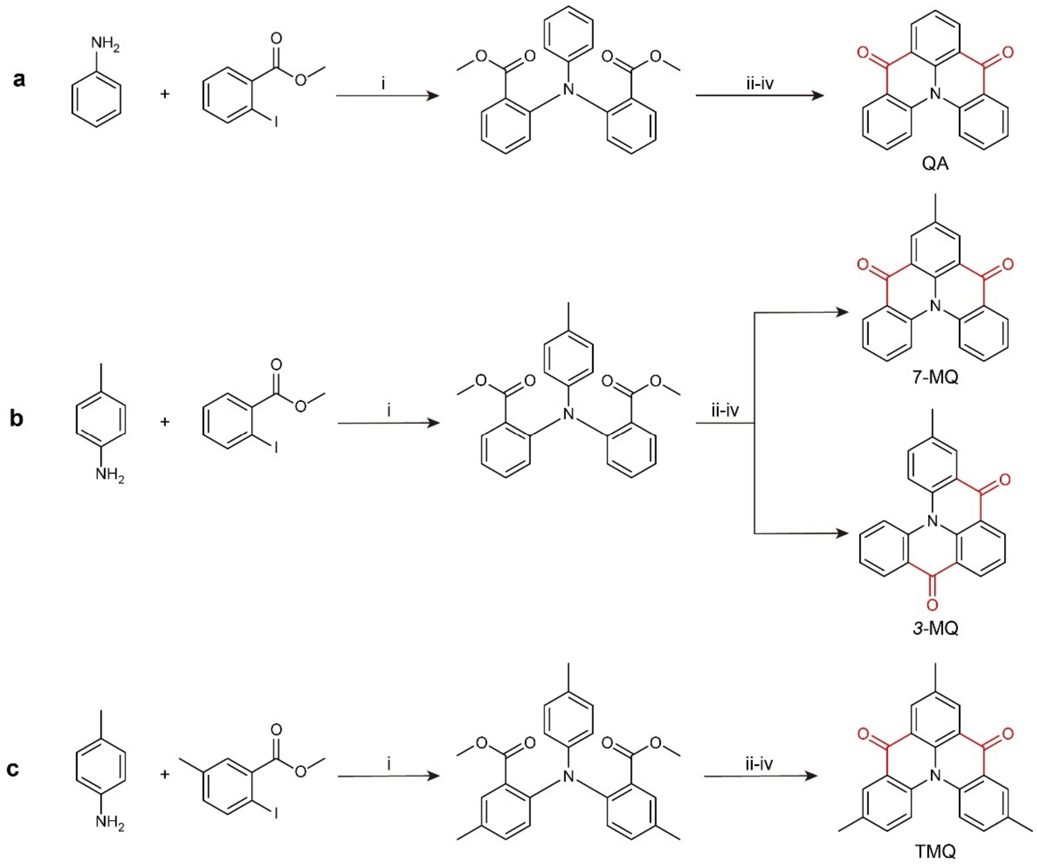

Supplementary Figure 2 | Synthetic routes of QA, 7-MQ, 3-MQ and TMQ molecules. (i) K_2CO_3, Cu/CuI, phenyl ether, 72 h, 453 K; (ii) KOH, methanol/H_2O (v/v)=1:1, 12 h, 373 K, 6 M HCl; (iii) $SOCl_2$, DMF (cat.), dichloromethane, 5 h; (iv) $AlCl_3$, 313 K, 48 h.

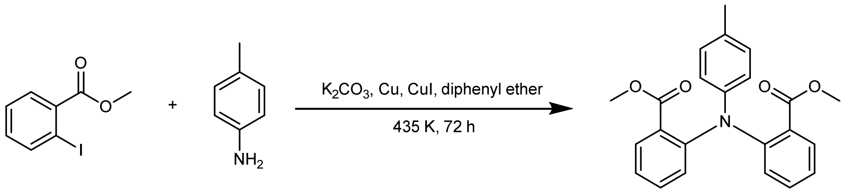

Dimethyl 2,2'-(*p*-tolylazanediyl)dibenzoate. To a 250 mL flask containing *p*-toluidine (5 g, 46.7 mmol), methyl 2-iodobenzoate (19 mL, 126 mmol), K_2CO_3 (17 g, 126 mmol), Cu (0.71 g, 11 mmol), CuI (0.45 g, 2.4 mmol) and diphenyl ether (40 mL) were added. The reaction mixture was stirred at 435 K for 72 h. After cooling to room temperature, the mixture was filtered. After the solvent was removed, the residue was purified by a column chromatography (petroleum ether/dichloromethane: 3:1) to afford 6.8 g faint yellow solid with a yield of 39%. ^{1}H NMR (400 MHz, DMSO-d_6): δ 7.53 (dd, J = 7.7 Hz, 2H), 7.48 (td, J = 7.8 Hz, 2H), 7.18 (t, J = 7.6 Hz, 2H), 7.05 (d, J = 8.0 Hz, 2H), 6.98 (d, J = 8.4 Hz, 2H), 6.57 (d, 2H), 3.29 (s, 6H), 2.20 (s, 3H). ^{13}C NMR (101 MHz, DMSO-d_6): δ 167.08, 145.98, 145.54, 132.59, 130.81, 130.53, 129.35, 128.06, 127.39, 123.85, 121.21, 51.49, 39.50, 20.28.

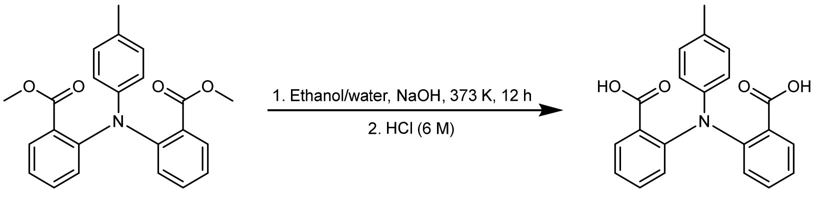

2,2'-(*p*-tolylazanediyl)dibenzoic acid. To a 100 mL bottle added 2, 2'-[(4-Methylphenyl)imino] bisbenzoicacid-dimethylester (2 g, 5.3 mmol), NaOH (1.1 g, 26.6 mmol), 25 mL water and 25 mL ethanol. The mixture was heated to 373 K for 12 h. After cooling to room temperature, the resulting acid was precipitated with concentrated HCl and dried under vacuum to give 1.8 g (81% yield). ^{1}H NMR (400 MHz, DMSO-d_6): δ 12.58 (s, 2H), 7.64 (dd, J = 7.7 Hz, 2H), 7.48 (td, J = 7.7 Hz, 2H), 7.20 (t, J = 7.5 Hz, 2H), 7.12 (d, J = 8.0 Hz, 2H), 6.89 (d, J = 8.5 Hz, 2H), 6.43 (d, J = 8.4 Hz, 2H), 2.16 (s, 3H). ^{13}C NMR (101 MHz, DMSO-d_6): δ 167.87, 146.11, 146.07, 132.67, 130.68, 129.28, 129.23, 129.03, 129.00, 124.33, 119.31, 20.22.

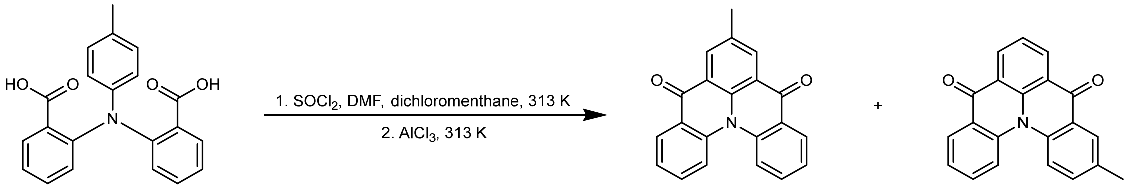

7-MQ 3-MQ

7-methylquinolino[3,2,1-de]acridine-5,9-dione (7-MQ) and 3-methylquinolino[3,2,1-de]acridine-5,9-dione (3-MQ). The 2,2'-(p-tolylazanediyl)dibenzoic acid (1 g, 2.9 mmol) was dispersed in dry dichloromethane (40 mL) with a few drops of DMF and then thionyl chloride (2 mL, 29 mmol) was added dropwise. The reaction was refluxed for 3 h and $AlCl_3$ (5.3 g, 40 mmol) was added. After refluxing for an additional 48 h, the reaction mixture was added dropwise to an aqueous solution of sodium hydroxide (≈ 1 M) and extracted with dichloromethane. The organic layer was dried over sodium sulfate and concentrated. The crude product was then purified by flash chromatography using dichloromethane/ petroleum ether (3/1, v/v) as the eluent to give yellow solid 7-MQ (330 mg, 36.8% yield) and 3-MQ (220 mg, 24.5% yield). **7-MQ**: ^{1}H NMR (400 MHz, $CDCl_3$): δ 8.55 (s, 2H), 8.49 (dd, J = 7.9 Hz, 2H), 8.14 (d, J = 8.6 Hz, 2H), 7.72 – 7.65 (m, 2H), 7.48 (t, J = 7.9 Hz, 2H), 2.61 (s, 3H). ^{13}C NMR (101 MHz, $CDCl_3$): δ 178.81, 139.87, 137.64, 133.92, 133.39, 132.80, 127.98, 126.52, 125.20, 123.45, 120.36, 21.00. Analysis (calcd., found for 7-MQ, C21H13NO2): C (81.01, 80.86), H(4.21, 3.96), N(4.50, 4.27). **3-MQ**: ^{1}H NMR (400 MHz, $CDCl_3$): δ 8.75 (d, J = 7.7 Hz, 2H), 8.50 (dd, J = 7.9 Hz, 1H), 8.29 (d, J = 1.7 Hz, 1H), 8.13 (d, J = 8.5 Hz, 1H), 8.04 (d, J = 8.7 Hz, 1H), 7.73 – 7.60 (m, 2H), 7.55 – 7.45 (m, 2H), 2.53 (s, 3H). ^{13}C NMR (101 MHz, $CDCl_3$): δ 178.90, 178.84, 140.05, 139.32, 137.84, 135.48, 134.08, 133.16, 133.06, 132.89, 127.98, 127.50, 126.52, 126.45, 125.22, 123.61, 123.60, 123.55, 120.46, 120.45, 21.07. Analysis (calcd., found for 3-MQ, $C_{21}H_{13}NO_2$): C (81.01, 81.07), H(4.21, 3.97), N(4.50, 4.29).

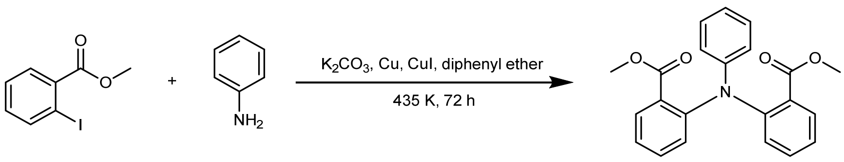

Dimethyl 2,2'-(phenylazanediyl)dibenzoate. The system route was the same as dimethyl 2,2'-(*p*-tolylazanediyl)dibenzoate. ^{1}H NMR (400 MHz, DMSO-d_6): δ 7.65 (d, J = 9.4 Hz, 2H), 7.44-7.38 (m, 2H), 7.20-7.15 (m, 4H), 7.12 (d, J = 8.6 Hz, 2H), 6.89 (t, J = 7.4 Hz, 1H), 6.76 (d, J = 7.6 Hz, 2H), 3.39 (s, 6H). ^{13}C NMR (101 MHz, DMSO-d_6): δ 167.04, 147.92, 145.70, 132.84, 130.61, 128.88, 128.47, 127.73, 124.33, 121.50, 120.37, 51.57, 39.50.

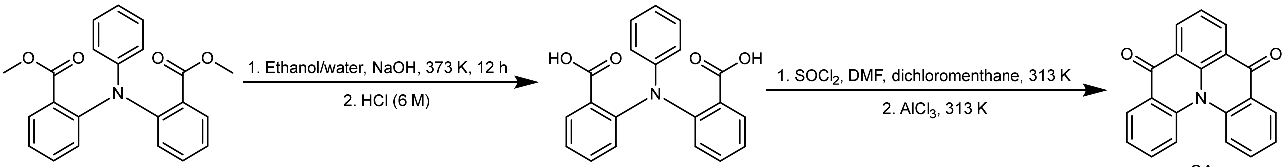

QA

Quinolino[3,2,1-*de*]acridine-5,9-dione (QA). The system routes were the same as 7-MQ and 3-MQ. ^{1}H NMR (400 MHz, $CDCl_3$): δ 8.75 (d, *J* = 7.7 Hz, 2H), 8.50 (dd, *J* = 7.9 Hz, 2H), 8.14 (d, *J* = 8.5 Hz, 2H), 7.74 – 7.62 (m, 3H), 7.50 (t, *J* = 8.0 Hz, 2H). ^{13}C NMR (101 MHz, $CDCl_3$): δ 178.81, 139.96, 139.45, 133.13, 128.01, 126.62, 125.42, 123.79, 123.60, 120.49, 77.25. Analysis (calcd., found for TMQ $C_{20}H_{11}NO_2$): C (80.80, 80.81), H (3.73, 3.82), N (4.71, 4.71).

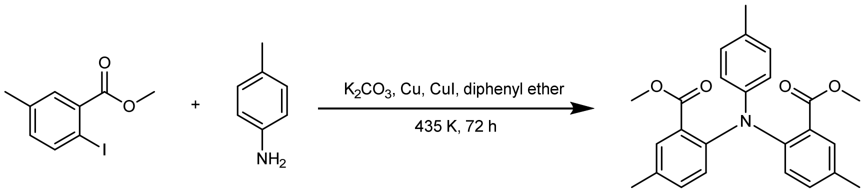

Dimethyl 6,6'-(*p*-tolylazanediyl)bis(3-methylbenzoate). The system route was the same as dimethyl 2,2'-(*p*-tolylazanediyl)dibenzoate. ^{1}H NMR (400 MHz, DMSO-d_6): δ 7.36 (s, 2H), 7.29 (d, *J* = 8.3 Hz, 2H), 6.99–6.89 (m, 4H), 6.48 (d, *J* = 7.1 Hz, 2H), 3.29 (d, *J* = 1.4 Hz, 6H), 2.29 (s, 6H), 2.18 (s, 3H). ^{13}C NMR (101 MHz, DMSO-d_6): δ 167.18, 145.92, 143.58, 133.32, 133.29, 130.70, 129.81, 129.20, 128.22, 127.36, 120.11, 51.52, 39.50, 20.19.

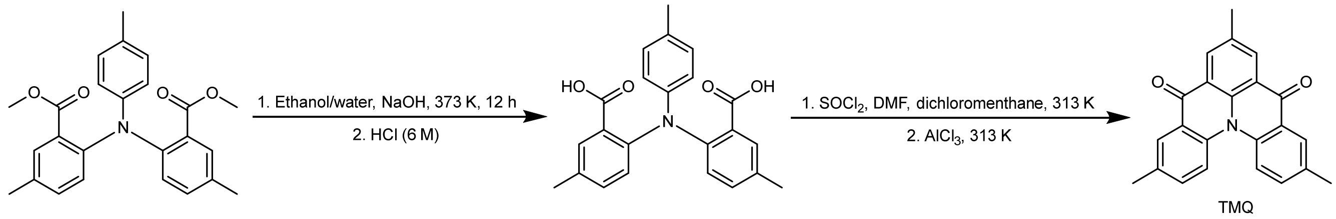

3,7,11-trimethylquinolino[3,2,1-*de*]acridine-5,9-dione (TMQ). The system routes were the same as 7-MQ and 3-MQ. ^{1}H NMR (400 MHz, $CDCl_3$): δ 8.56 (s, 2H), 8.28 (s, 2H), 8.03 (d, *J* = 8.6 Hz, 2H), 7.49 (d, *J* = 8.5 Hz, 2H), 2.61 (s, 3H), 2.52 (s, 6H). ^{13}C NMR (101 MHz, $CDCl_3$): δ 178.59, 137.53, 137.10, 134.70, 133.62, 133.20, 133.06, 127.11, 125.95, 123.09, 119.99, 20.72, 20.68. Analysis (calcd., found for TMQ $C_{23}H_{17}NO_2$): C (81.40, 81.24), H (5.05, 5.11), N (4.13, 4.08).

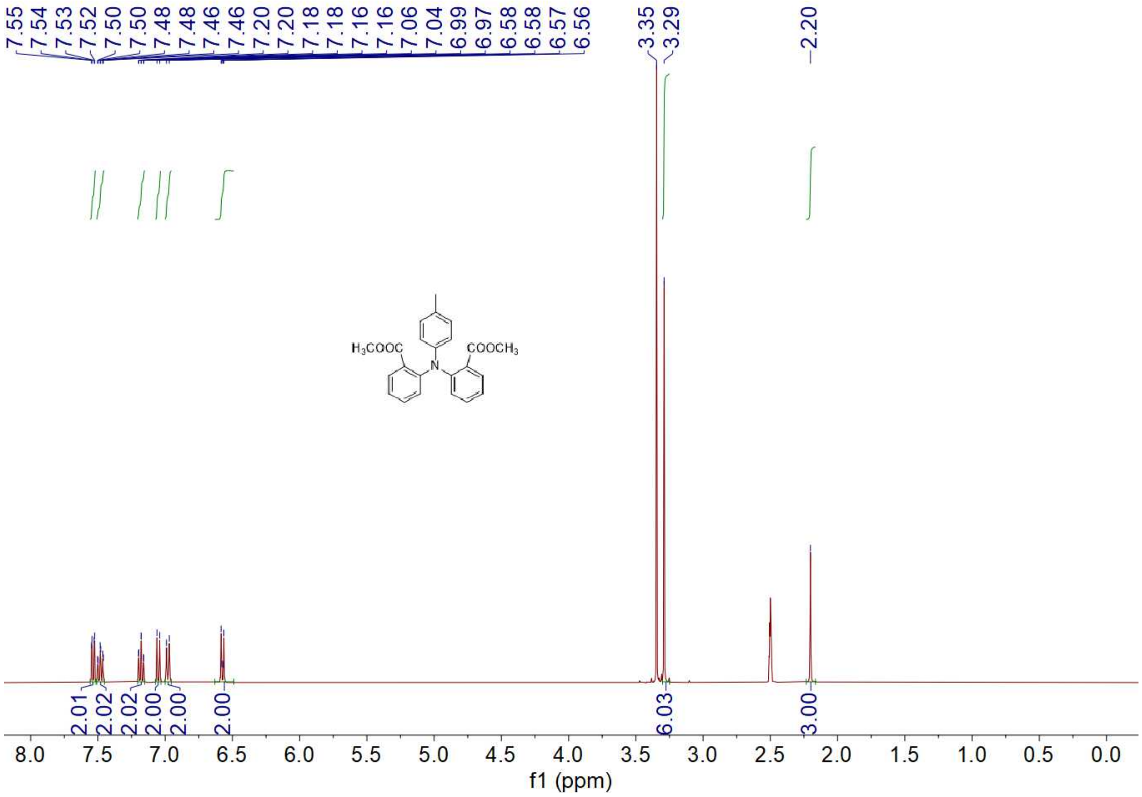

Supplementary Figure 3 | ^{1}H NMR spectrum of dimethyl 2,2'-(p-tolylazanediyl)dibenzoate in DMSO-d_6.

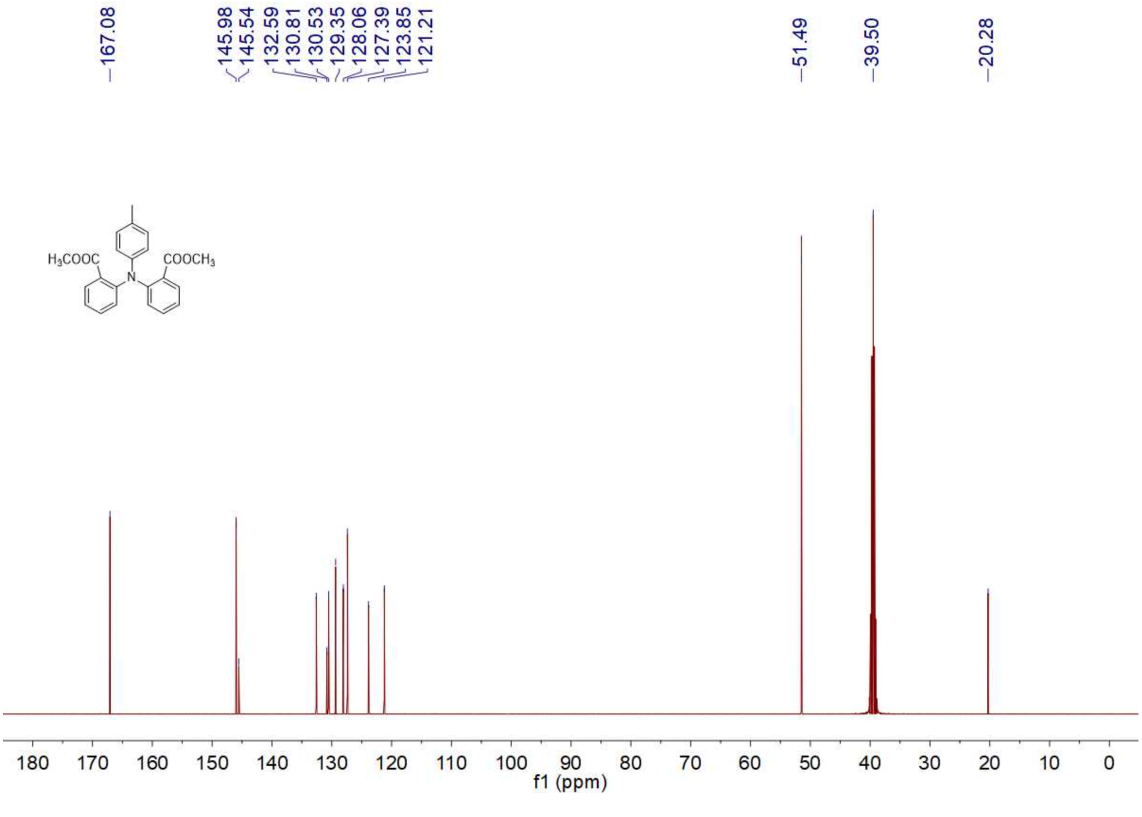

Supplementary Figure 4 | ^{13}C NMR spectrum of dimethyl 2,2'-(p-tolylazanediyl)dibenzoate acid in DMSO-d_6.

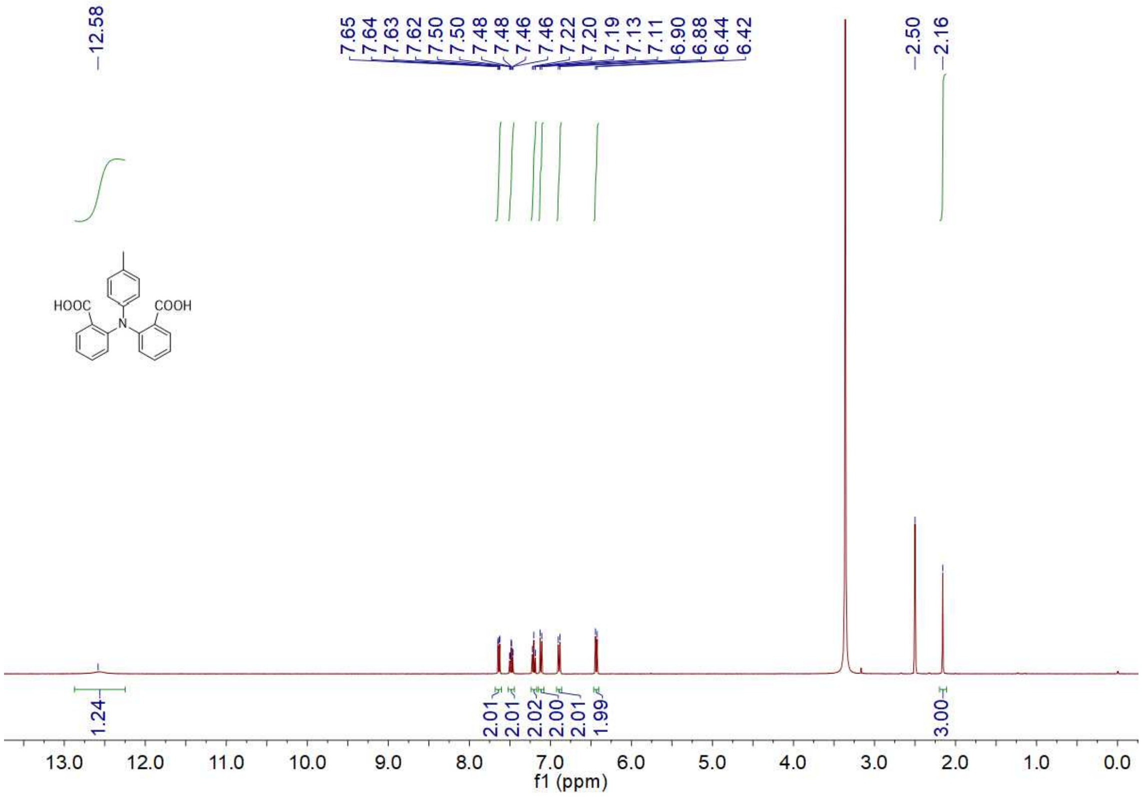

Supplementary Figure 5 | ^{1}H NMR spectrum of 2,2'-(p-tolylazanediyl)dibenzoic acid in DMSO-*d*$_6$.

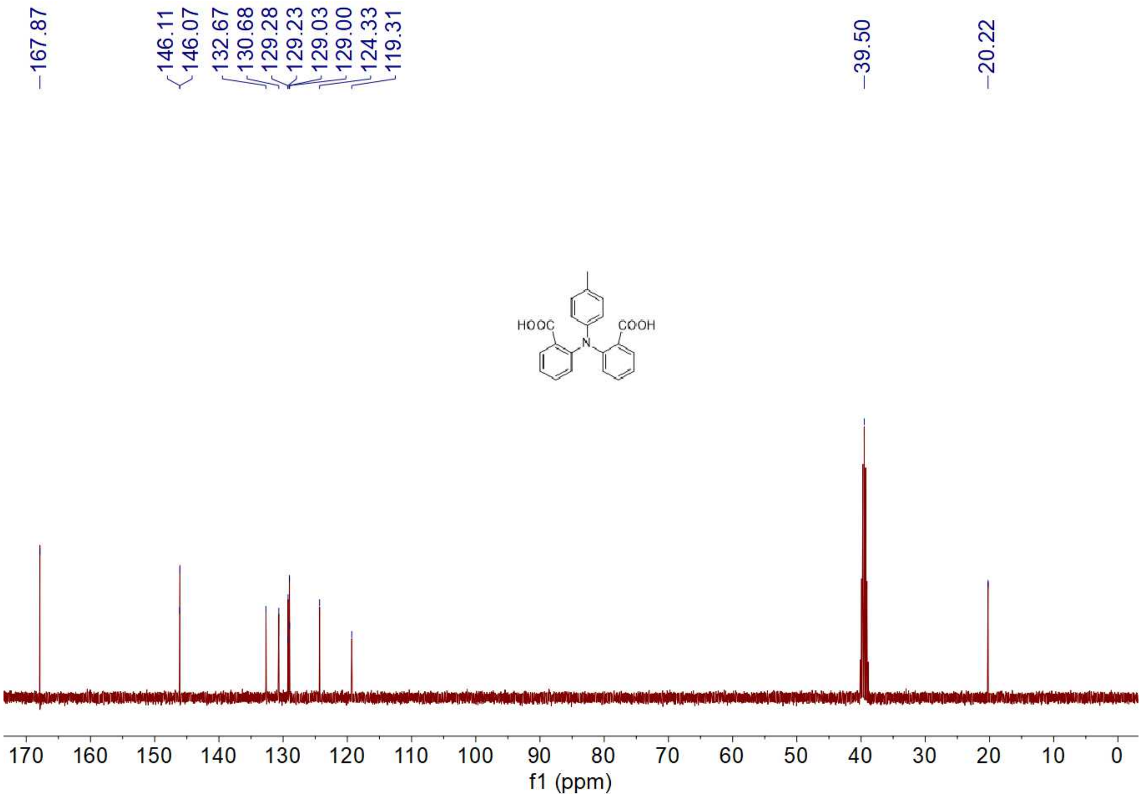

Supplementary Figure 6 | ^{13}C NMR spectrum of 2,2'-(p-tolylazanediyl)dibenzoic acid in DMSO-*d*$_6$.

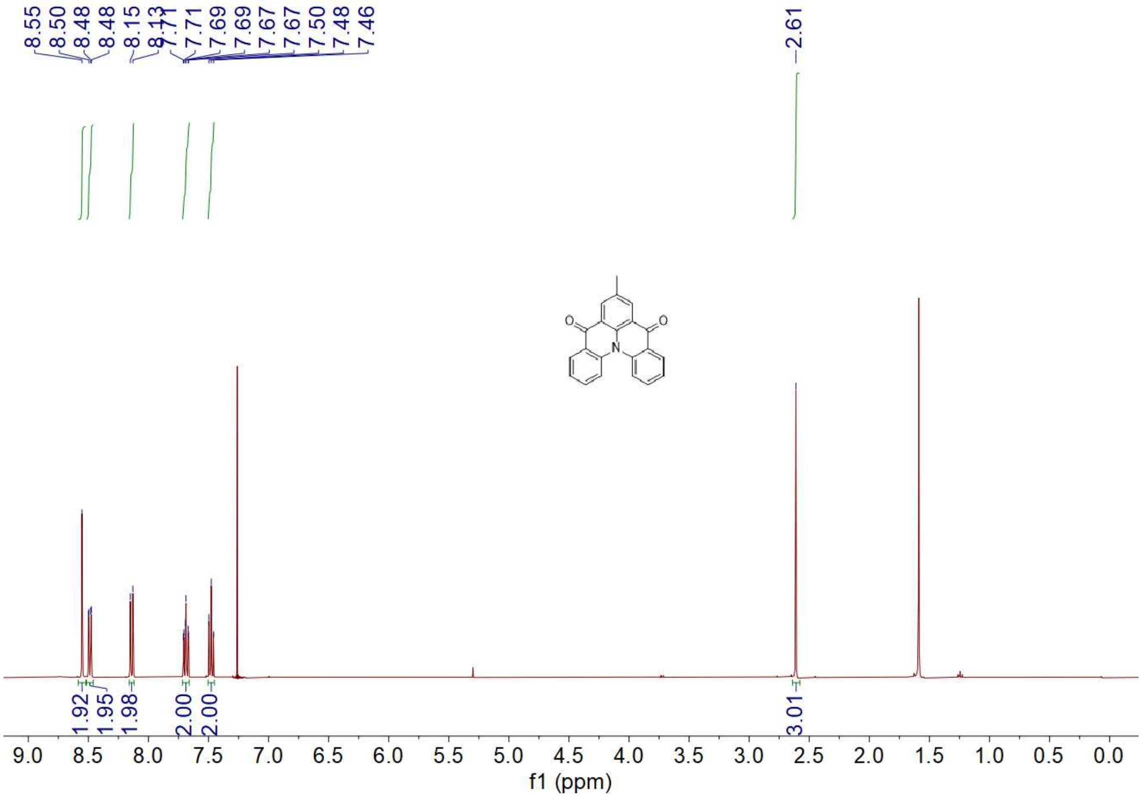

Supplementary Figure 7 | ^{1}H NMR spectrum of 7-MQ in $CDCl_3$-d_1.

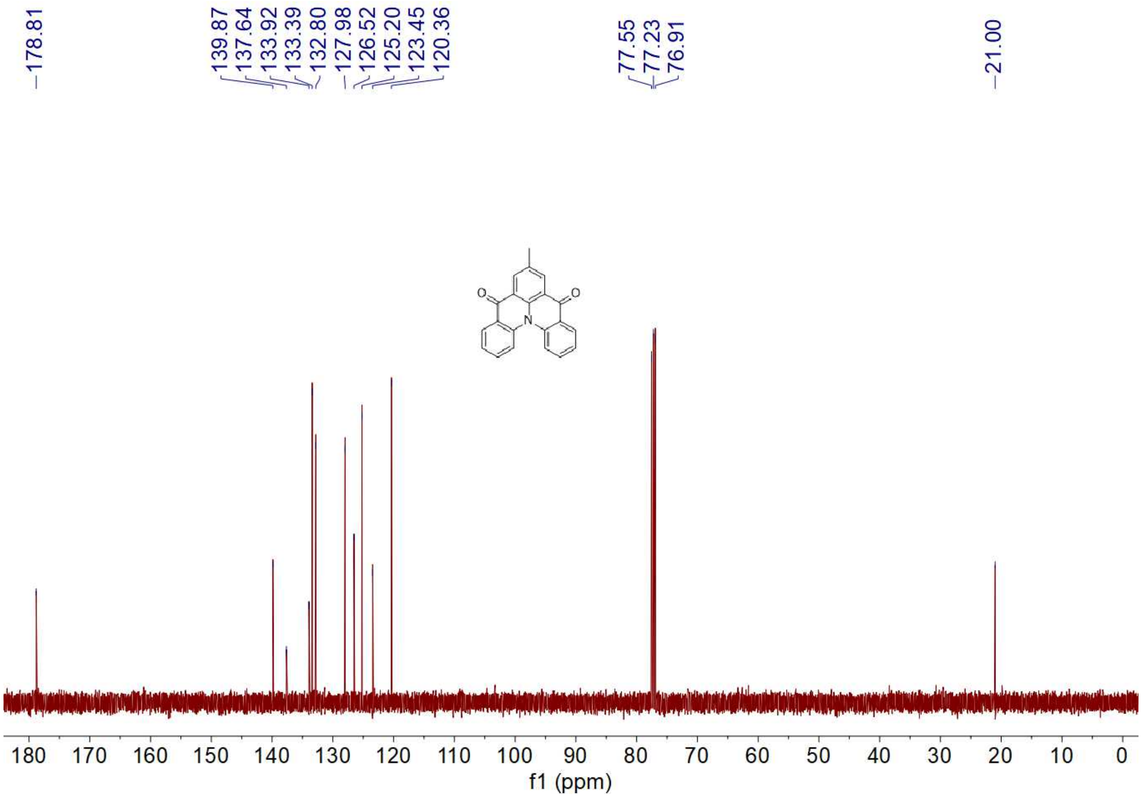

Supplementary Figure 8 | ^{13}C NMR spectrum of 7-MQ in $CDCl_3$-d_1.

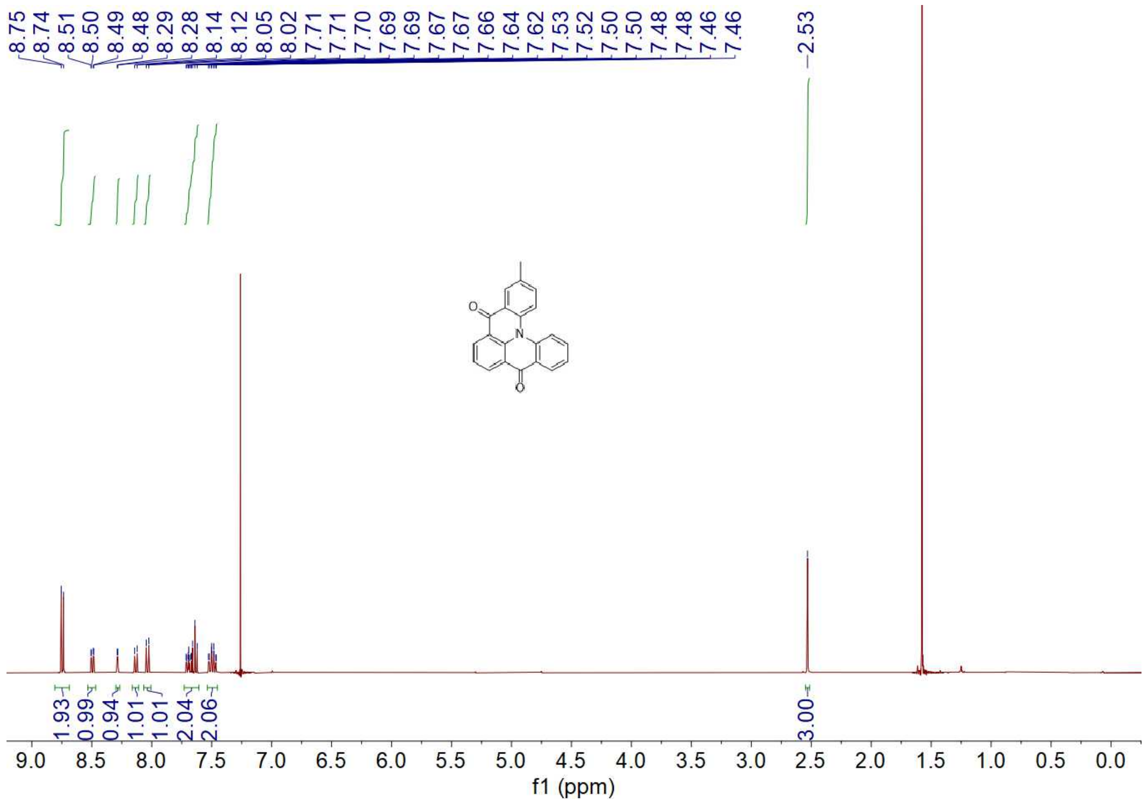

Supplementary Figure 9 | ^{1}H NMR spectrum of 3-MQ in $CDCl_3$-*d*$_1$.

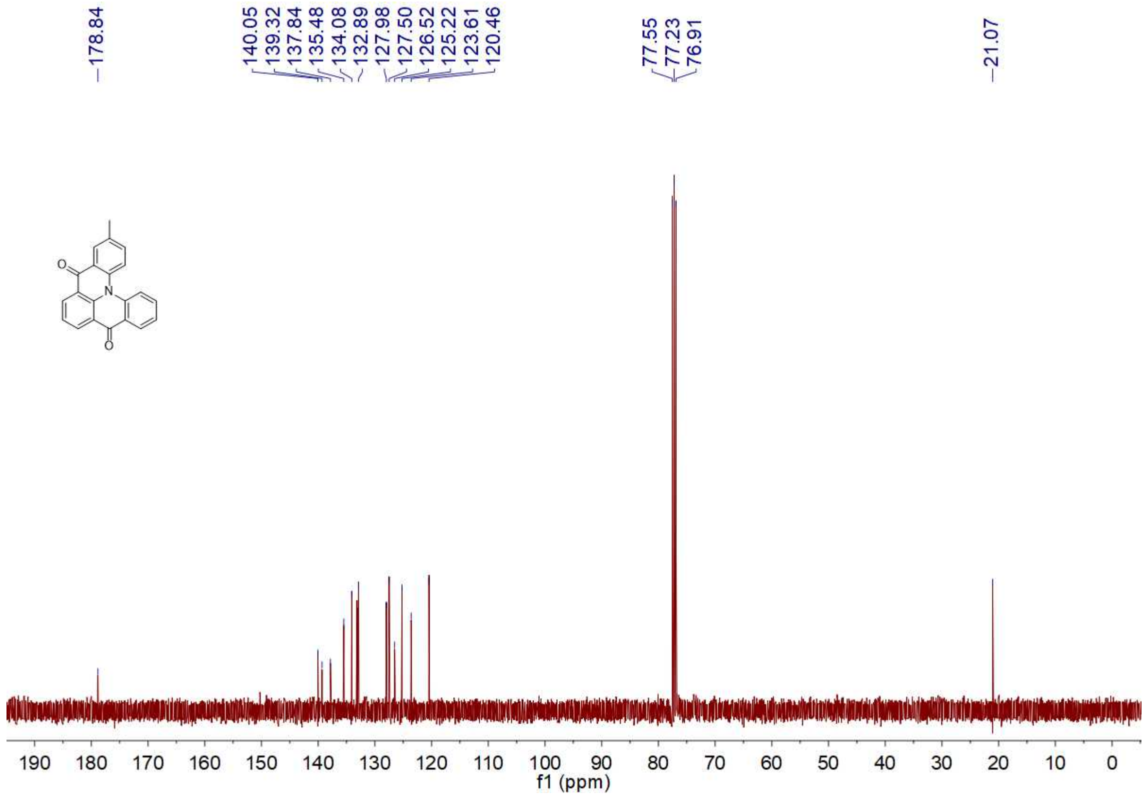

Supplementary Figure 10 | ^{13}C NMR spectrum of 3-MQ in $CDCl_3$-*d*$_1$.

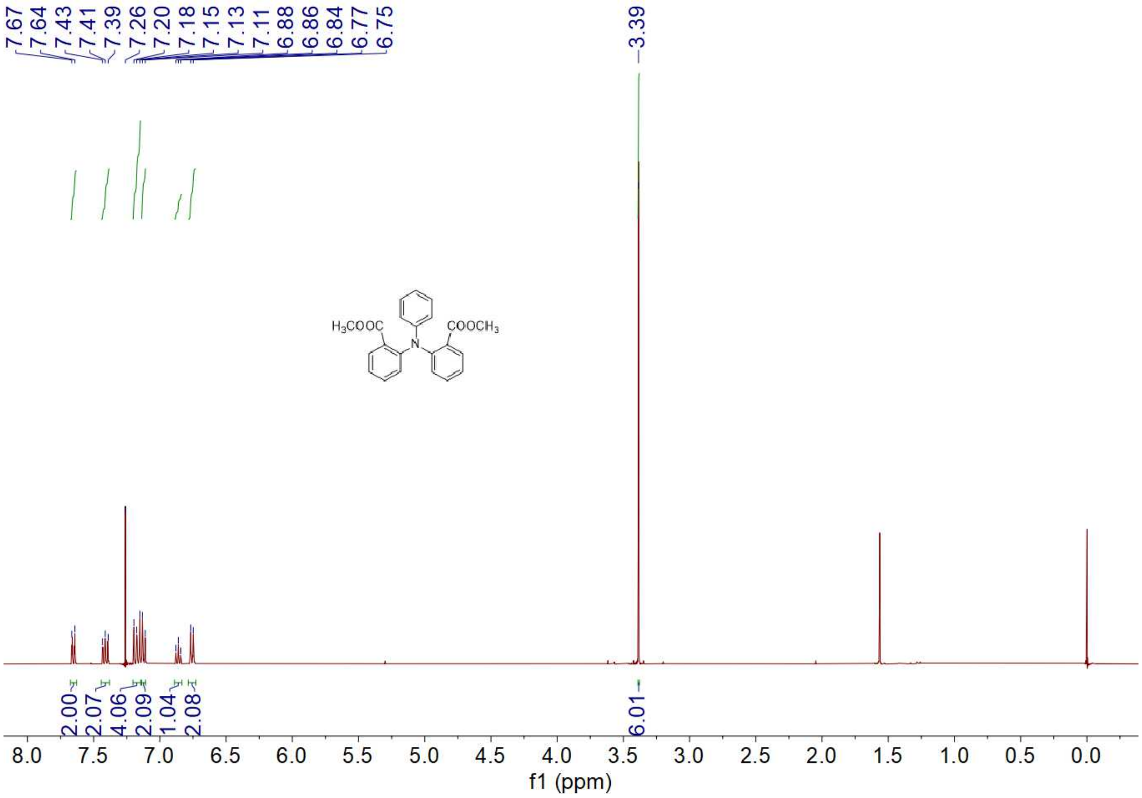

Supplementary Figure 11 | ^{1}H NMR spectrum of dimethyl 2,2'-(phenylazanediyl)dibenzoate in $CDCl_3$-*d*$_1$.

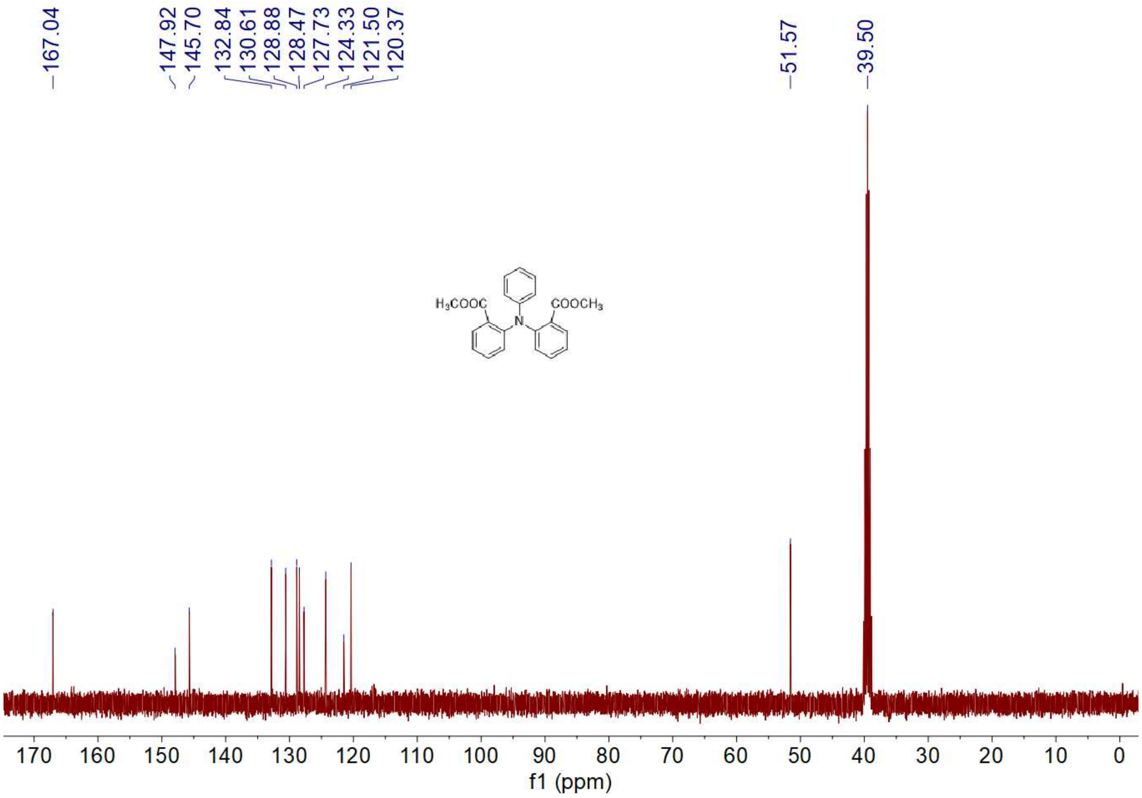

Supplementary Figure 12 | ^{13}C NMR spectrum of dimethyl 2,2'-(phenylazanediyl)dibenzoate in DMSO-*d*$_6$.

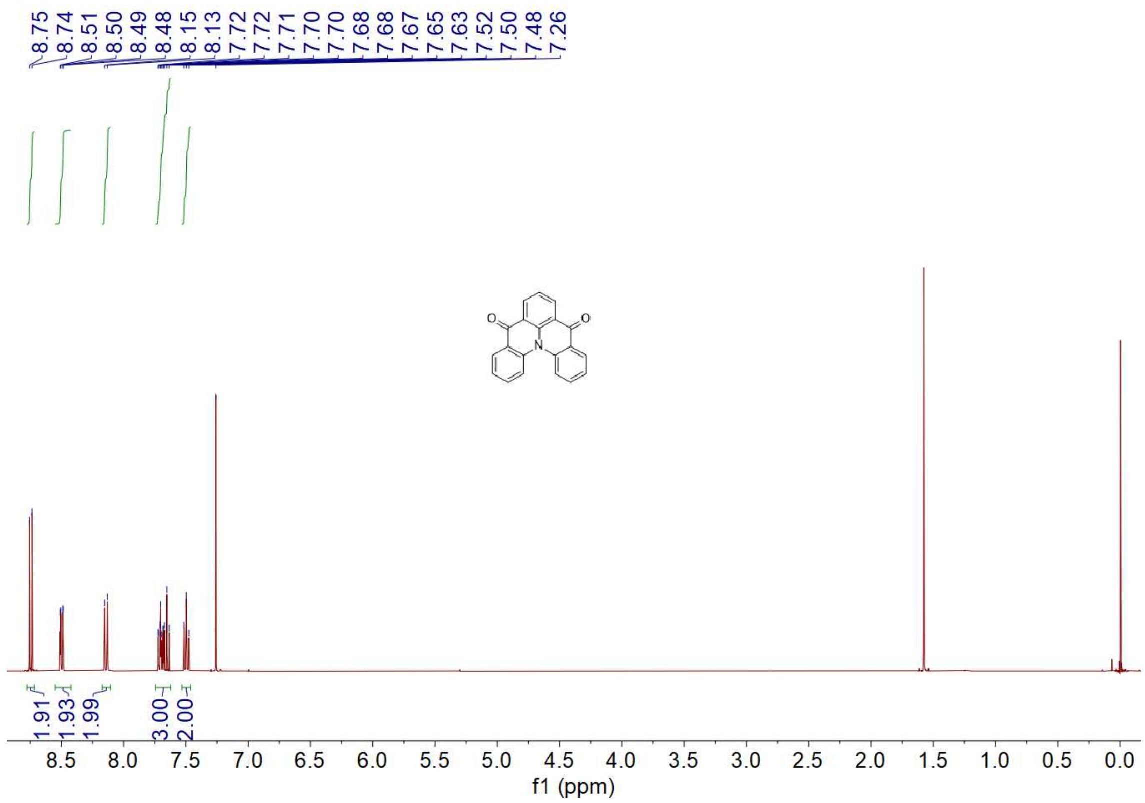

Supplementary Figure 13 | ^{1}H NMR spectrum of QA in $CDCl_3$-*d*$_1$.

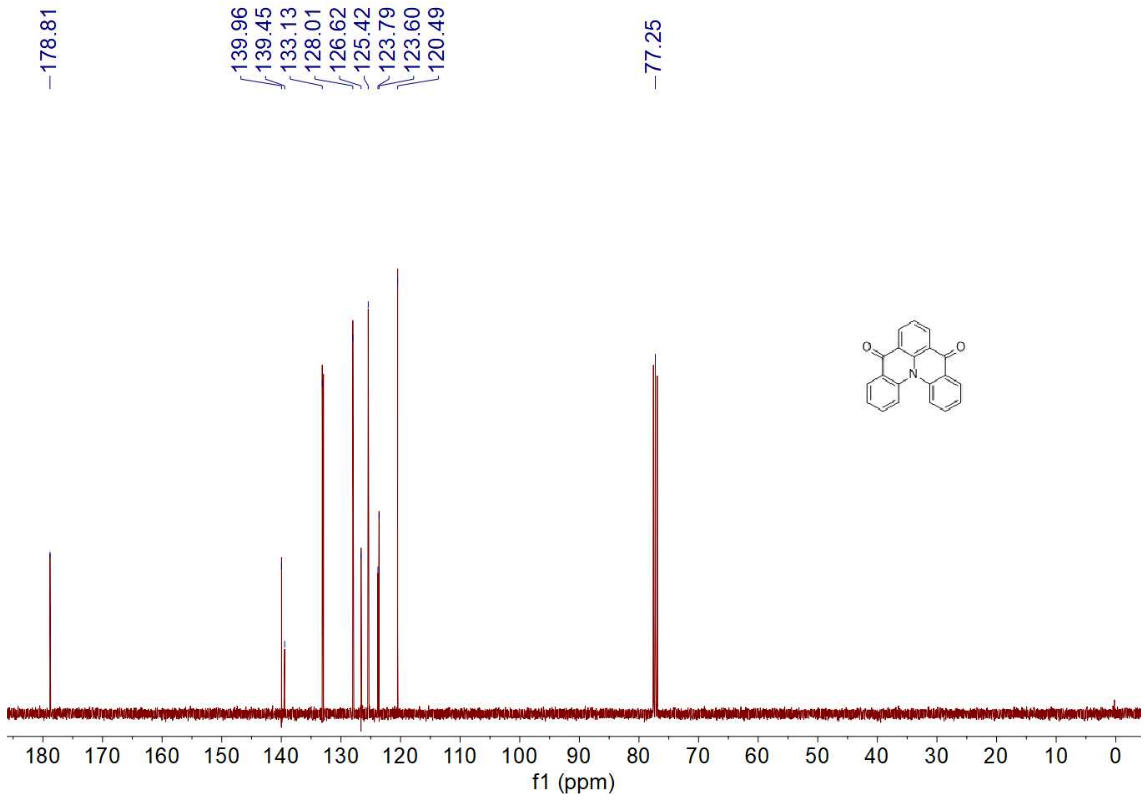

Supplementary Figure 14 | ^{13}C NMR spectrum of QA in $CDCl_3$-*d*$_1$.

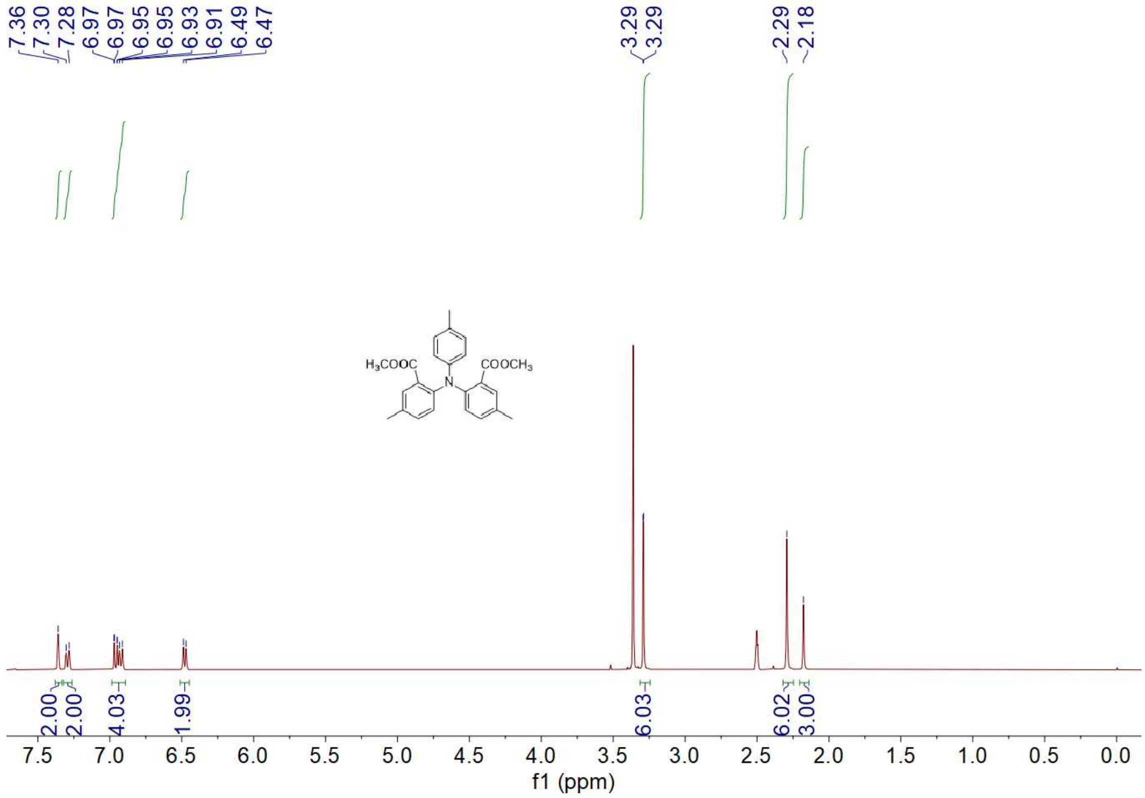

Supplementary Figure 15 | ^{1}H NMR spectrum of dimethyl 6,6'-(*p*-tolylazanediyl)bis(3-methylbenzoate) in DMSO-*d*$_6$.

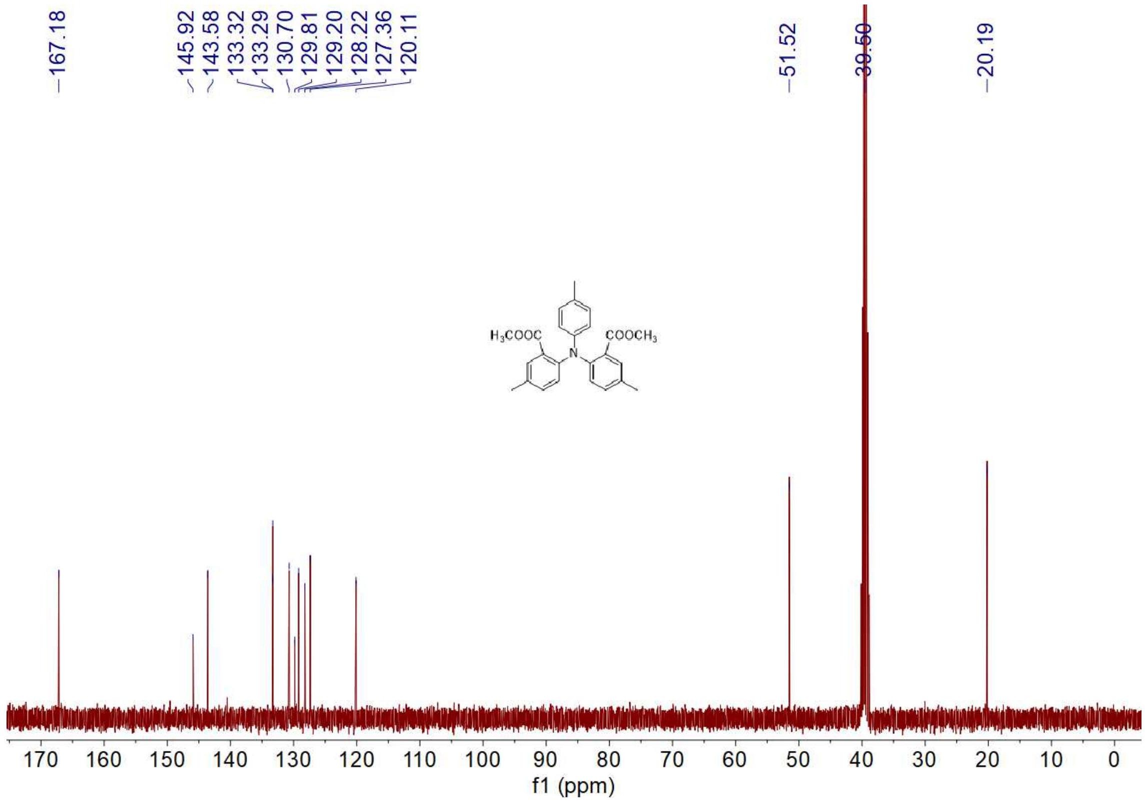

Supplementary Figure 16 | ^{1}H NMR spectrum of dimethyl 6,6'-(*p*-tolylazanediyl)bis(3-methylbenzoate) in DMSO-*d*$_6$.

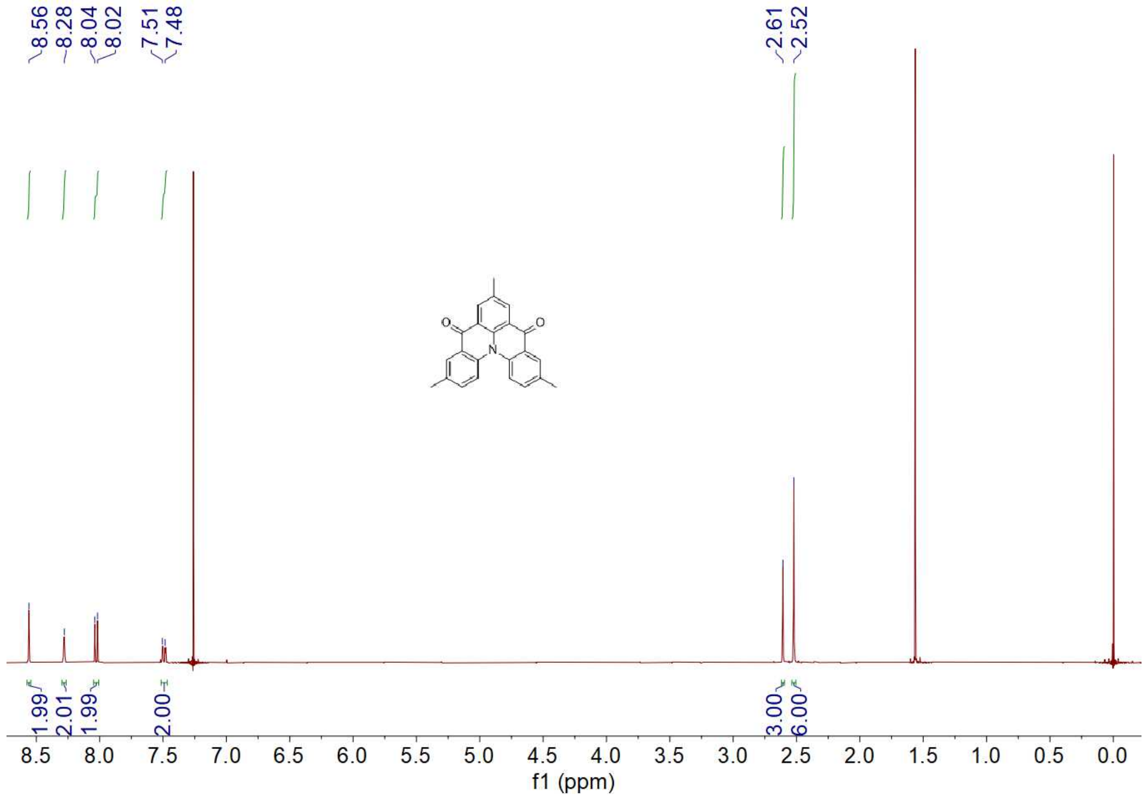

Supplementary Figure 17 | ^{1}H NMR spectrum of TMQ in CDCl$_3$-*d*$_1$.

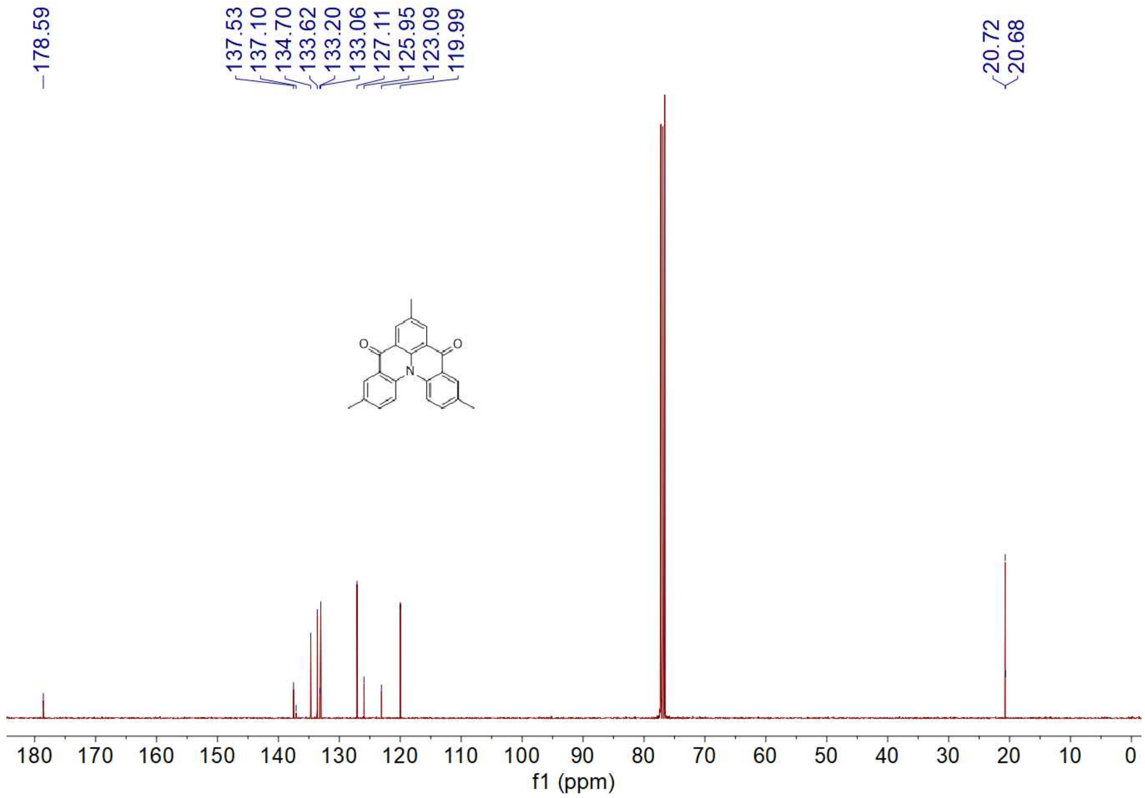

Supplementary Figure 18 | ^{13}C NMR spectrum of TMQ in CDCl$_3$-*d*$_1$.

Suppl. Note 4. Photophysical properties of organic emitters in solid and solution

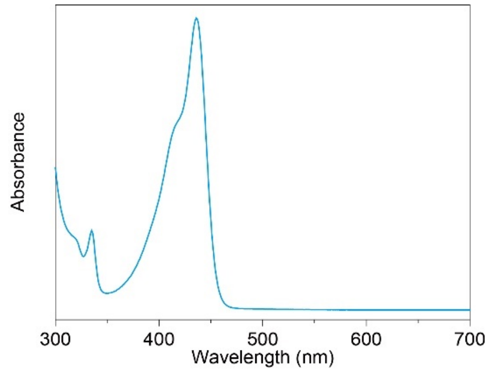

Supplementary Figure 19 | Absorption spectrum of the 7-MQ molecule in *m*-THF solution under ambient conditions. Source data are provided as a Source Data file.

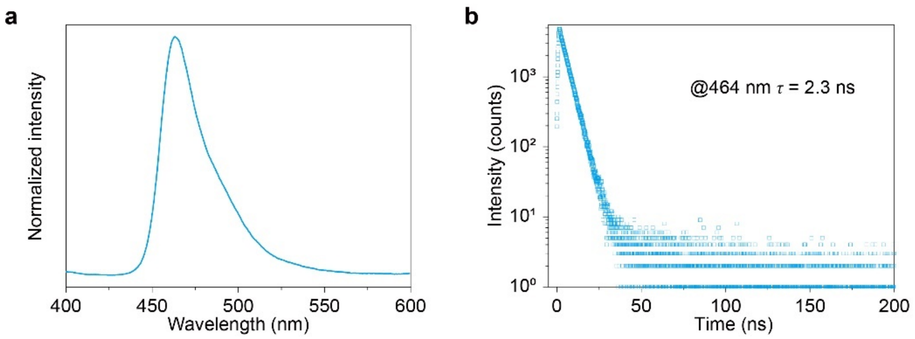

Supplementary Figure 20 | Photoluminescence properties of 7-MQ molecule (1 × 10^{-5} M) in *m*-THF solution at room temperature. a, Normalized steady-state PL spectrum. **b**, Lifetime decay curve. Source data are provided as a Source Data file.

Supplementary Table 2 | Lifetimes of the 7-MQ molecule in *m*-THF solution (1 × 10^{-5} M) at room temperature.

Wavelength (nm)	τ (ns)	Proportion (%)
464	4.3	100

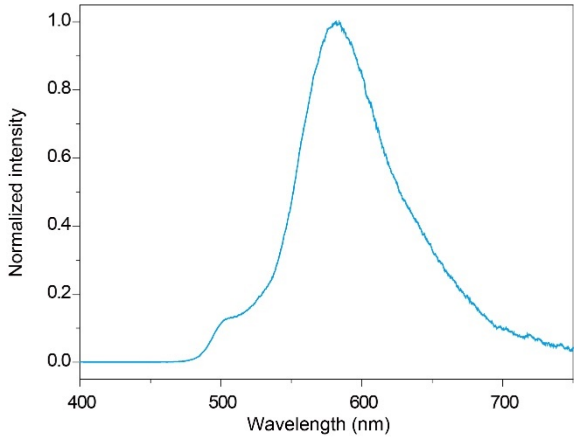

Supplementary Figure 21 | Normalized steady-state photoluminescence spectrum of the 7-MQ solid. The

excitation wavelength is 380 nm. Source data are provided as a Source Data file.

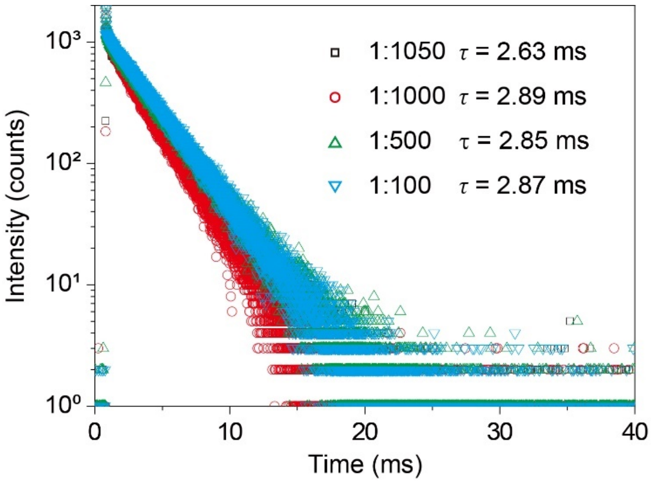

Supplementary Figure 22 | Lifetime decay curves of the 7-MQ in BP matrix with various doped ratio under ambient conditions. The excitation wavelength is 380 nm. Source data are provided as a Source Data file.

Supplementary Table 3 | Lifetimes of the 7-MQ molecule in BP matrix with different doping ratio under ambient conditions.

Ratio	τ (ms)	Proportion (%)
1:1050	2.63	100
1:1000	2.89	100
1:500	2.85	100
1:100	2.87	100

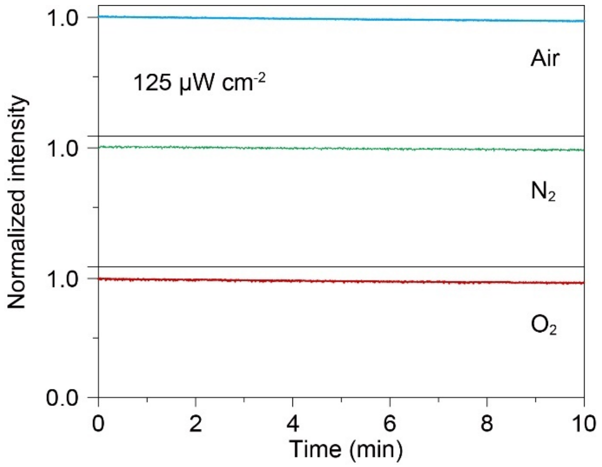

Supplementary Figure 23 | Phosphorescence stability under various atmosphere. Phosphorescence intensity at 495 nm of the 7-MQ phosphor in BP matrix with a ratio of 1:1000 as a function of time under various atmosphere. Power of excitation light is 125 μW cm^{-2}. Source data are provided as a Source Data file.

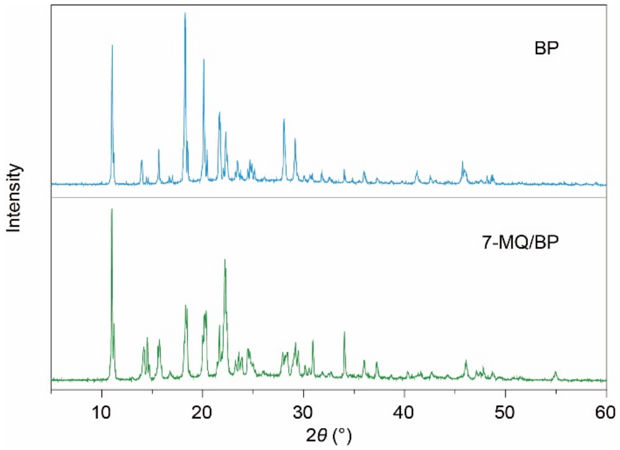

Supplementary Figure 24 | Experimental PXRD patterns of the 7-MQ/BP and pure BP solid under ambient conditions. The doping ratio is 1:1000 for the 7-MQ/BP. Source data are provided as a Source Data file.

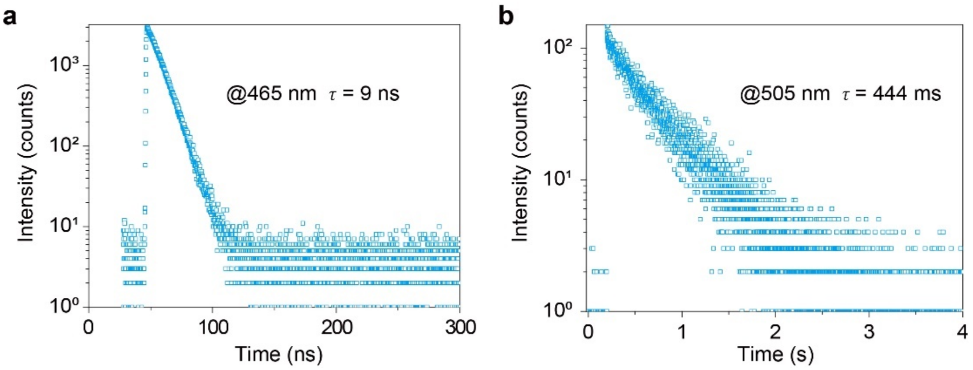

Supplementary Figure 25 | Lifetime delay curves of the emission at 465 (a) and 505 (b) nm for the 7-MQ molecule in *m*-THF (1 × 10^{-5} M) solution at 77 K. Source data are provided as a Source Data file.

Supplementary Table 4 | Lifetimes of the 7-MQ molecule in *m*-THF (1 × 10^{-5} M) solution at 77 K.

Wavelength (nm)	τ	Proportion (%)
465	9.9 ns	100
505	444.5 ms	100

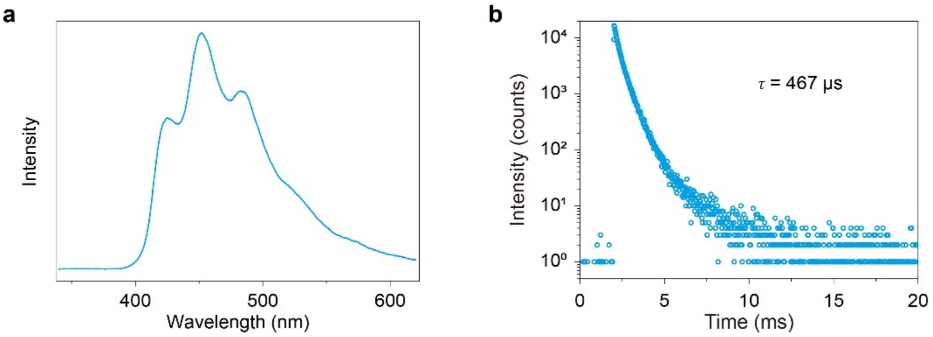

Supplementary Figure 26 | Photophysical properties of BP crystal under ambient conditions. a, Steady-state PL spectrum. **b**, Lifetime decay curve of the emission at 450 nm. Source data are provided as a Source Data file.

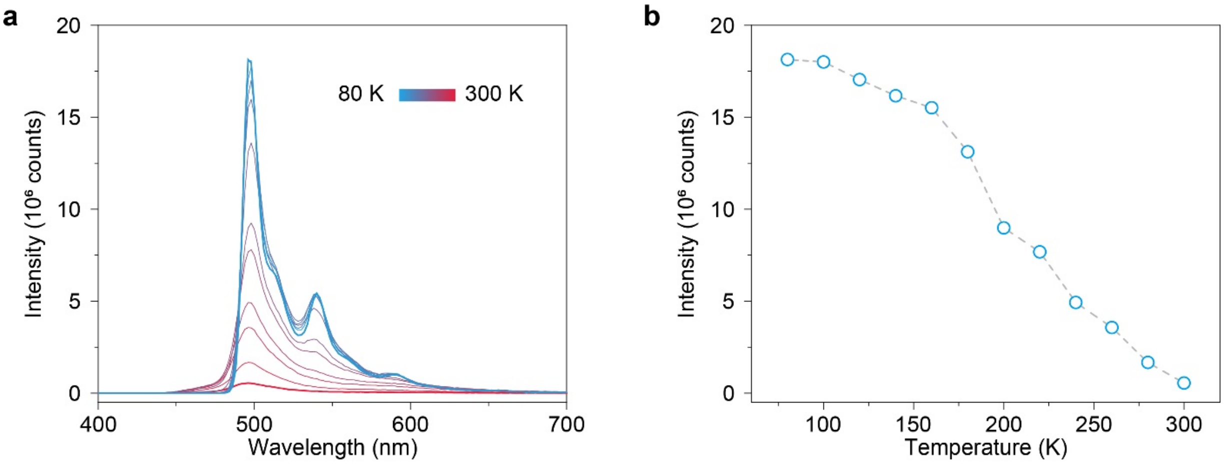

Supplementary Figure 27 | Temperature-dependent delayed photoluminescence spectra (a) and emission intensity (b) of the 7-MQ in the BP matrix. The delay time is 8.0 ms. Source data are provided as a Source Data file.

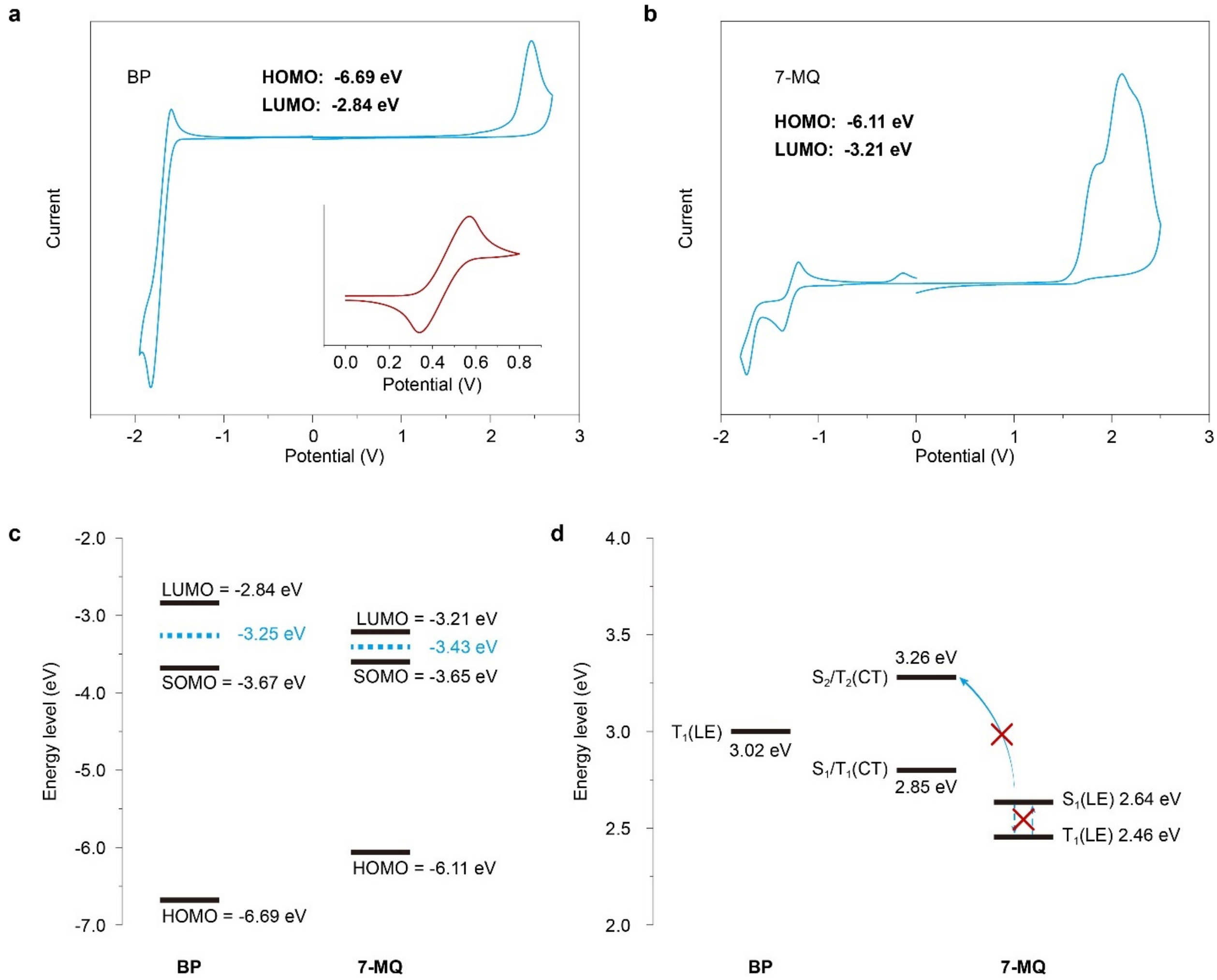

Supplementary Figure 28 | Energy alignment for BP and 7-MQ. Cyclic voltammetry curves of the BP (**a**) and 7-MQ (**b**) molecules. Orbital energy alignment (**c**) and schematic of the state energy alignment (**d**) for BP and 7-MQ. The blue dashed lines represent the equalized energy levels for the LUMO and SOMO without considering the electron exchange energy. Source data are provided as a Source Data file.

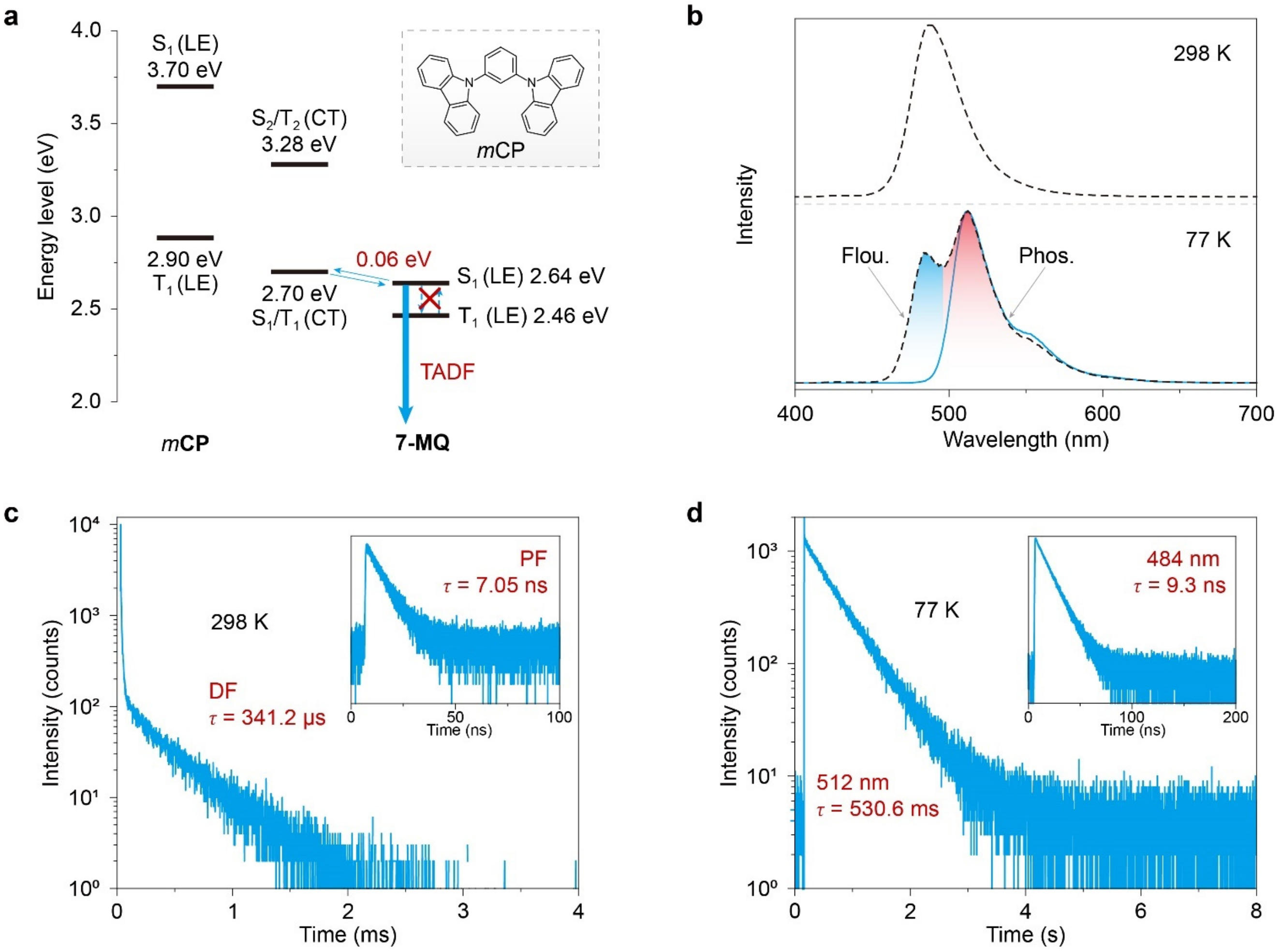

Supplementary Figure 29 | Photophysical properties of 7-MQ in the *m*CP. a, Schematic of the energy alignment for the 7-MQ in *m*CP. **b**, Normalized steady-state and delay photoluminescence spectra (with a delay gate of 8.0 ms) of the 7-MQ/*m*CP at 298 and 77 K. **c**, **d**, Lifetime time decay curves of the 7-MQ/*m*CP at 298 and 77 K. Note that PF and DF are the prompt fluorescence and delay fluorescence, respectively. Source data are provided as a Source Data file.

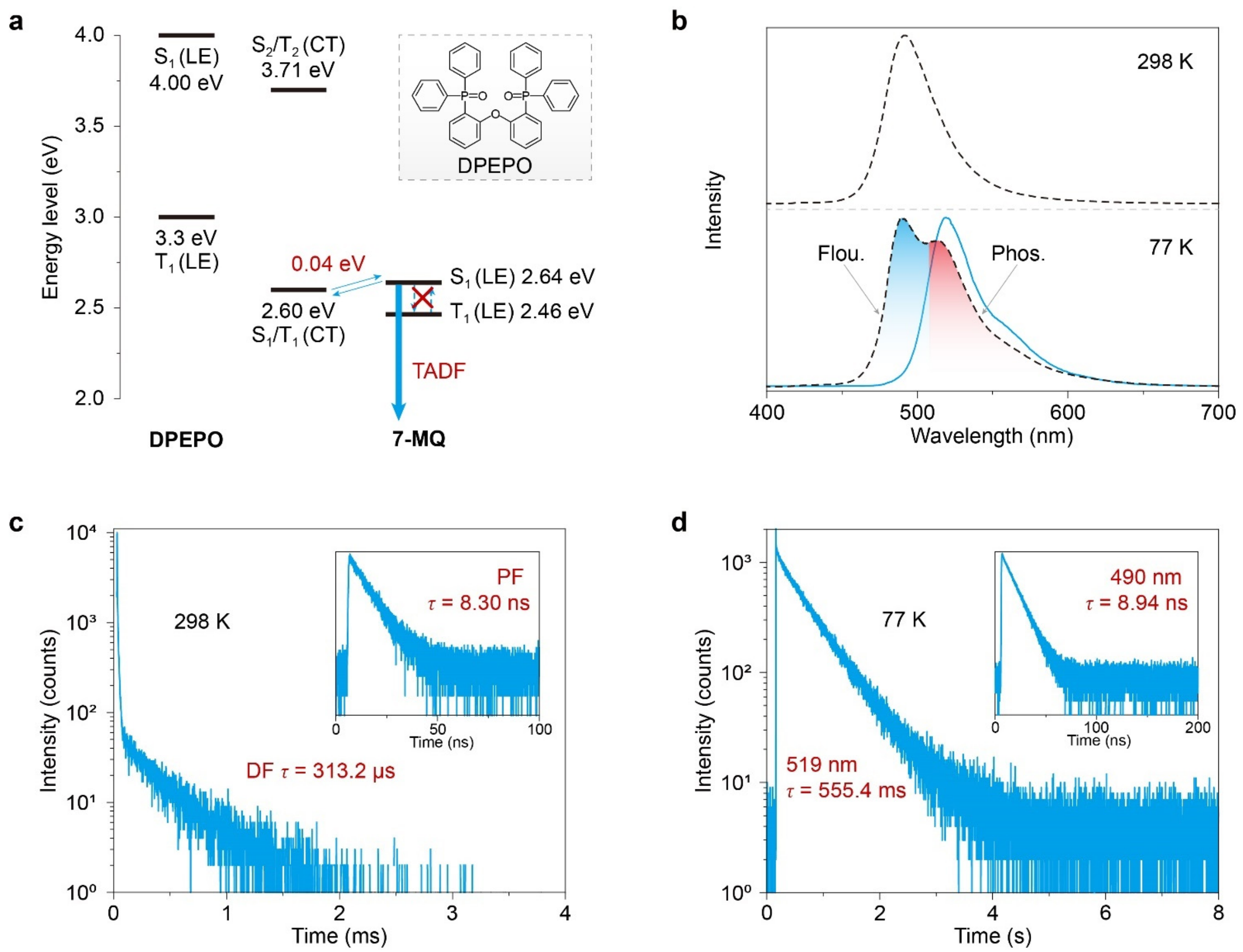

Supplementary Figure 30 | Photophysical properties of 7-MQ in the DPEPO. a, Schematic of the energy alignment for the 7-MQ in DPEPO. **b**, Normalized steady-state and delay photoluminescence spectra (with a delay gate of 8.0 ms) of the 7-MQ/ DPEPO at 298 and 77 K. **c**, **d**, Lifetime time decay curves of the 7-MQ/ DPEPO at 298 and 77 K. Note that PF and DF are the prompt fluorescence and delay fluorescence, respectively. Source data are provided as a Source Data file.

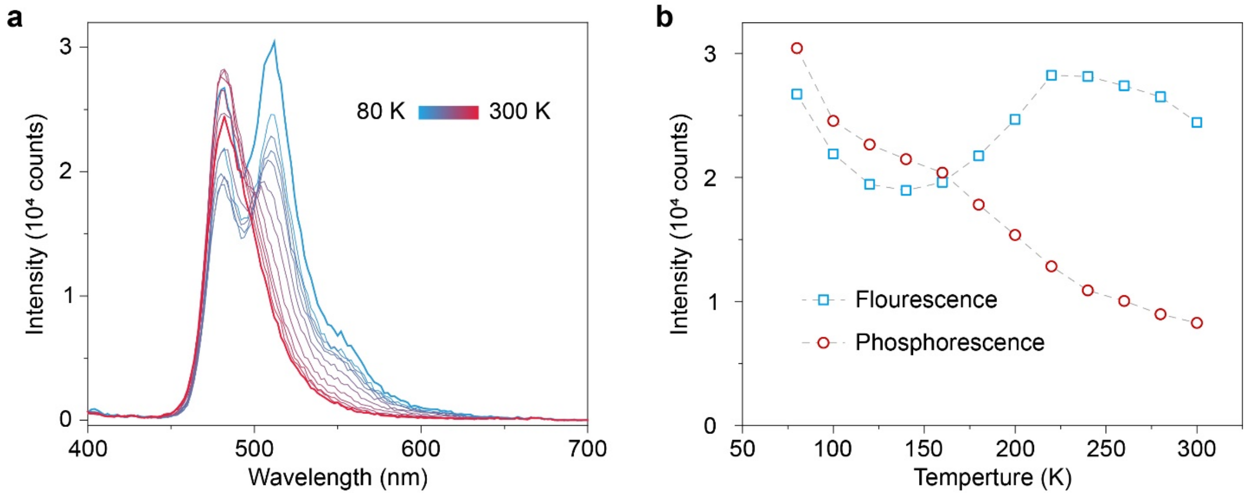

Supplementary Figure 31 | Temperature-dependent emission properties of the 7-MQ in the mCP matrix. Temperature-dependent steady-state photoluminescence spectra (**a**) and intensity (**b**) of the 7-MQ in the mCP matrix**.** Source data are provided as a Source Data file.

Suppl. Discussion 1. Analysis of the host-guest energy levels

To demonstrate the difference between phosphorescence and TADF of the 7-MQ molecule, *m*CP and DPEPO host molecules were chosen. The energy levels of *m*CP and DPEPO were referred from previous work[251]. There exist thermally accessible processes for the 7-MQ in *m*CP and DPEPO matrices due to the small energy gap between the S_1(CT) in *m*CP (0.06 eV) and DPEPO (0.04 eV) and S_1(LE) of the 7-MQ molecule, respectively (Supplementary Figs. 29a and 30a). From Supplementary Figs. 29b and 30b, it is found that the emission decay curves exhibit double-exponential for the 7-MQ in *m*CP and DPEPO matrices under ambient conditions, indicating there are TADF components for emission. There is an obvious difference in the steady state spectra of the 7-MQ in *m*CP and DPEPO matrices at 298 and 77 K (Supplementary Figs. 29c and 30c). Besides the fluorescent emission with peaks at 484 and 490 nm, new emission band appears for the 7-MQ in *m*CP and DPEPO after cooled to 77 K. The emission lifetimes are up to 530.6 and 555.4 ms for the 7-MQ in *m*CP and DPEPO (Supplementary Figs. 29d and 30d), respectively, which were ascribed to phosphorescence. When the 7-MQ was doped into BP matrix, there existed different emission properties. As shown in Figure 3c in the revised manuscript, the steady-state and delay spectra of the 7-MQ in BP are overlap. There is only one emission band with a peak at 495 nm. As temperature decreased from room temperature to 77 K, the emission intensity increases but no change for emission band (Figure 3a in the manuscript). Therefore, we reasoned that the emission band at around 495 nm corresponds to phosphorescence in BP matrix.

Subsequently, we measured the temperature-dependent steady-state photoluminescence spectra of the 7-MQ doped in *m*CP solid. As shown in Supplementary Fig. 31, there is a significant difference in emission intensity for the fluorescence (484 nm) and phosphorescence (512 nm) peaks. The intensity of the fluorescence peak shows an upward tendency from 140 to 220 K, while phosphorescence intensity continues to decrease in the range of 80-300 K. The upward tendency of fluorescence is attributed to the thermally activated exciton transition from the triplet to singlet states. The temperature-dependent phosphorescence intensity of the 7-MQ in the *m*CP shows a similar tendency to the 7-MQ in the BP matrix.

In summary, from the above experimental results and analysis, we believe that the emission of 7-MQ at 495 nm is phosphorescence in the BP matrix.

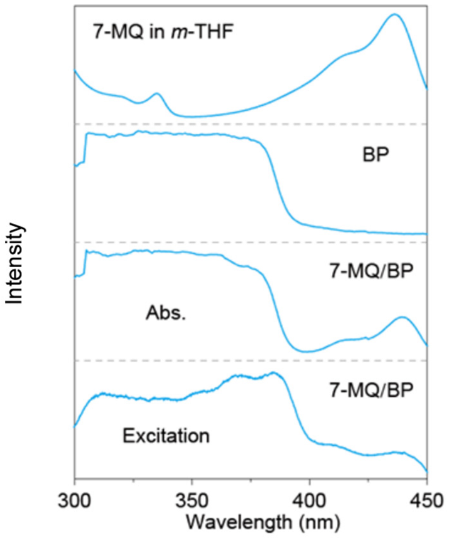

Supplementary Figure 32 | Absorption and excitation spectra for the 7-MQ in *m*-THF, BP solid and 7-MQ/BP phosphor. From top to bottom, the spectra are the curves of absorption of 7-MQ in *m*-THF and BP solid, 7-MQ/BP, as well as the excitation spectrum of 7-MQ/BP, respectively. Source data are provided as a Source Data file.

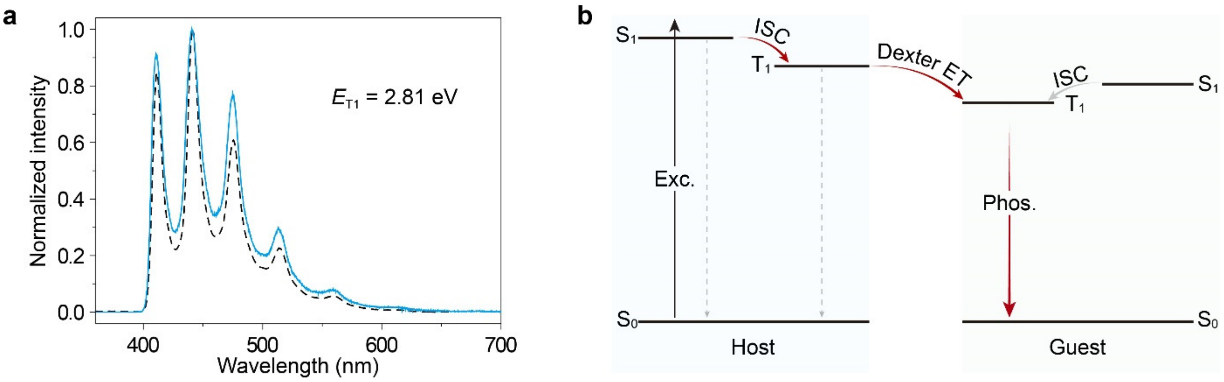

Supplementary Figure 33 | Proposed energy transfer processes for MR molecules in the BP matrix. a, Normalized steady-state and delay spectra of the BP molecules in m-THF solution at 77 K. **b**, Proposed energy transfer processes for 7-MQ/BP. Note that E_{T1}, Exc., ISC, ET and Phos. are the abbreviation of the energy level of lowest excited triplet state, intersystem crossing, energy transfer and phosphorescence, respectively. Source data are provided as a Source Data file.

Suppl. Discussion 2. Energy transfer between the host and the guest component

There is an overlap between the steady-state and delay spectra for the 7-MQ molecule in the BP matrix at room temperature and 77 K, indicating that the triplet excitons of the 7-MQ molecule can be efficiently generated for phosphorescence in the BP matrix (Supplementary Fig. 33a). Herein, we reasoned that efficient triplet-triplet energy transfer between the host and guest molecules and enhanced spin-orbit coupling of the 7-MQ molecule in the BP matrix play critical roles in boosting phosphorescence. BP is a typical molecule with pure phosphorescence in solid state (Supplementary Fig. 26), which is beneficial to enhance phosphorescence emission with Dexter energy transfer (Supplementary Fig. 33b).

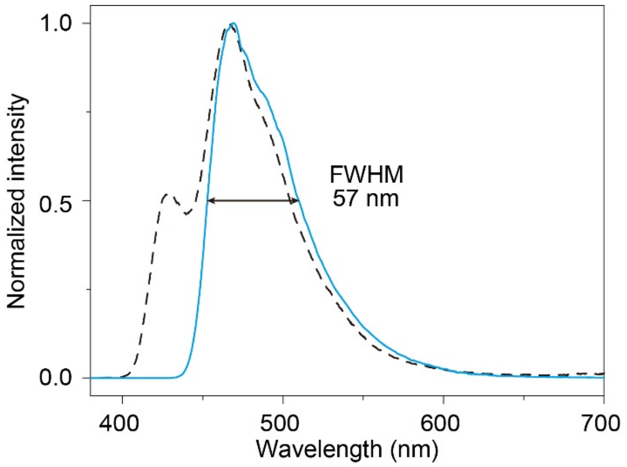

Supplementary Figure 34 | Normalized steady-state PL (dash line) and phosphorescence (solid line) spectra of the 2,2'-(p-tolyazanediyl)dibenzoate in *m*-THF (1 × 10^{-5} M) solution at 77K. The FWHM of the emitter is 57 nm. Source data are provided as a Source Data file.

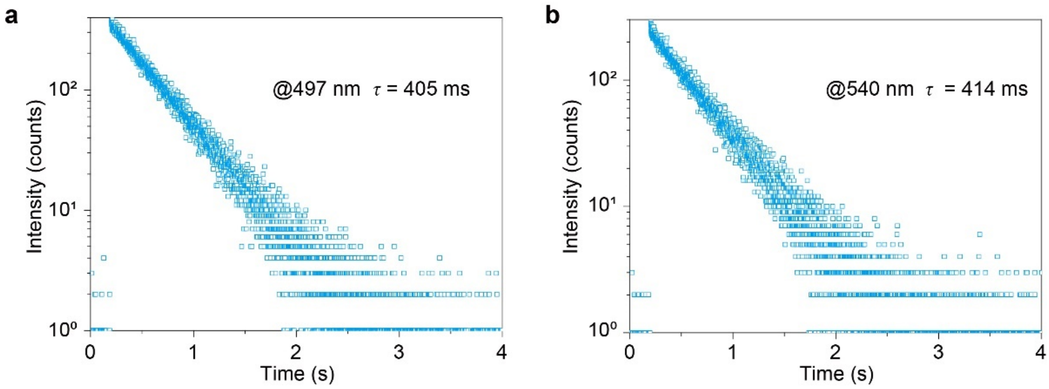

Supplementary Figure 35 | Lifetime decay curves of the emission at 495 (a) and 540 (b) nm for the 7-MQ in BP matrix (1:1000) at 77 K. Source data are provided as a Source Data file.

Supplementary Table 5 | Phosphorescence lifetimes of the 7-MQ in BP matrix at 77 K.

Wavelength (nm)	τ	Proportion (%)
497	405	100
540	414	100

Suppl. Note 5. Photophysical properties of the extended emitters

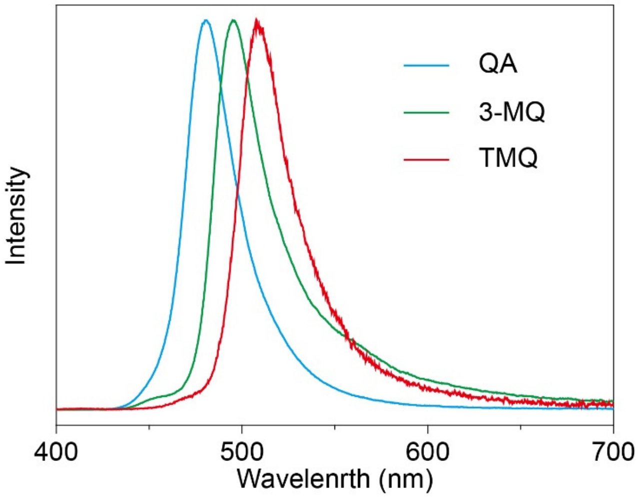

Supplementary Figure 36 | Normalized steady-state PL spectra of the QA, 3-MQ and TMQ in the BP matrix under ambient conditions. Source data are provided as a Source Data file.

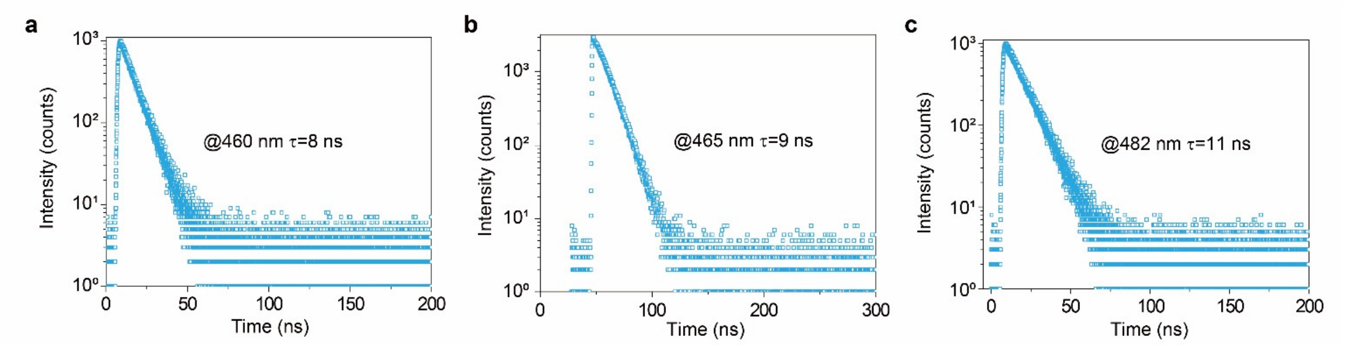

Supplementary Figure 37 | Lifetime decay curves of the QA (a), 3-MQ (b) and TMQ (c) molecules in *m*-THF solution at 77 K. The emission wavelengths are 460, 465 and 482 nm for the QA, 3-MQ and TMQ, respectively. Note that the excitation wavelength is 375 nm UV light. Source data are provided as a Source Data file.

Supplementary Table 6 | Lifetimes of the QA, 3-MQ and TMQ molecules in *m*-THF solution at 77 K.

Name	Wavelength (nm)	τ(ns)	Proportion (%)
QA	460	8.0	100
3-MQ	465	9.0	100
TMQ	482	10.6	100

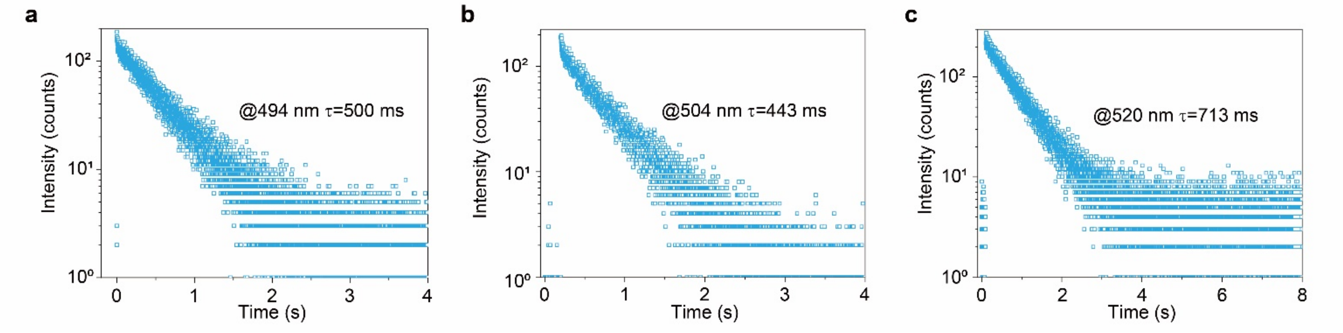

Supplementary Figure 38 | Long-lived lifetime decay curves of the QA (a), 3-MQ (b) and TMQ (c) molecules in *m*-THF solution (1 × 10^{-5} M) at 77 K. The emission wavelengths 494, 443 and 520 nm for the QA, 3-MQ and TMQ in *m*-THF matrix at 77 K, respectively. Source data are provided as a Source Data file.

Supplementary Table 7 | Lifetimes of the QA, 3-MQ and TMQ in *m*-THF solution at 77 K.

Name	Wavelength (nm)	τ(ms)	Proportion (%)
QA	494	499.8	100
3-MQ	504	443.1	100
TMQ	520	713.0	100

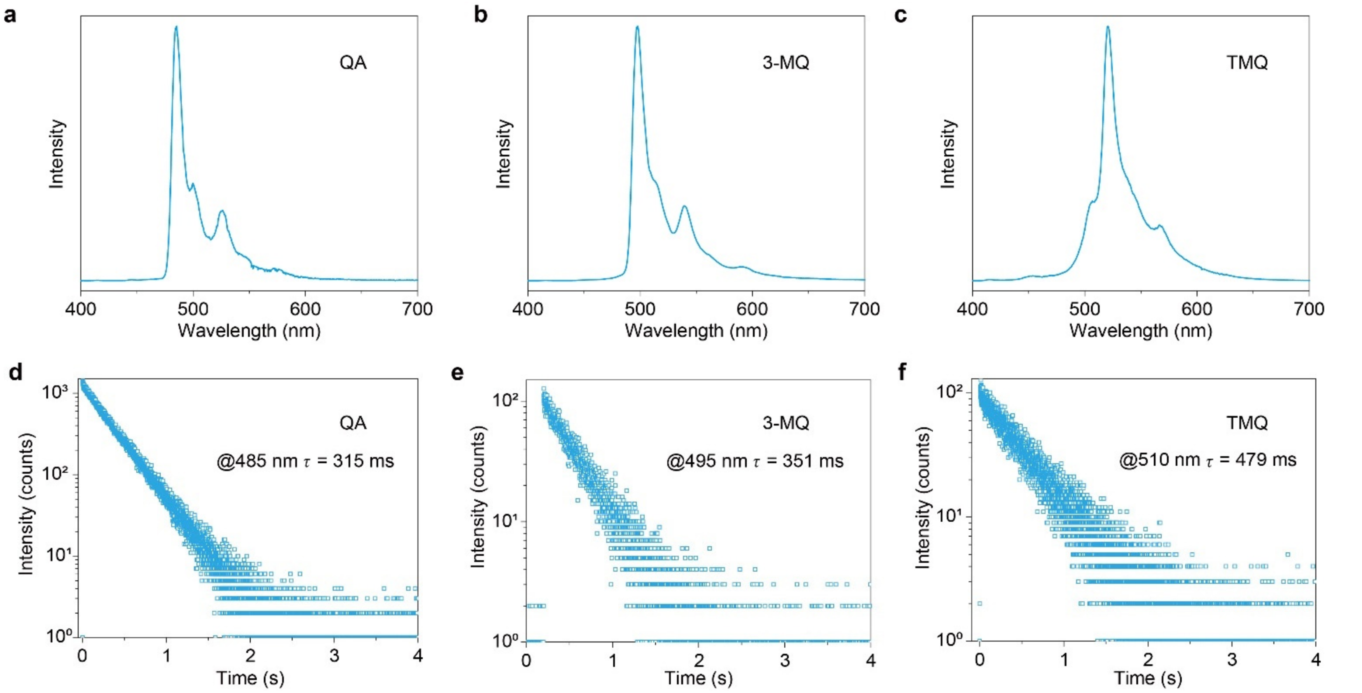

Supplementary Figure 39 | Photophysical properties of the QA, 3-MQ and TMQ molecules in BP matrix at 77 K. a-c, Normalized phosphorescence spectra of QA (**a**), 3-MQ (**b**) and TMQ (**c**) in BP matrix. **d-f**, Profiles of lifetime curves for the QA (**d**), 3-MQ (**e**) and TMQ (**f**) in BP matrix at 77 K. Source data are provided as a Source Data file.

Supplementary Table 8 | Lifetimes of the QA, 3-MQ and TMQ in BP matrix at 77 K.

Wavelength (nm)	τ(ms)	Proportion (%)
485	315.5	100
495	351.0	100
510	479.7	100

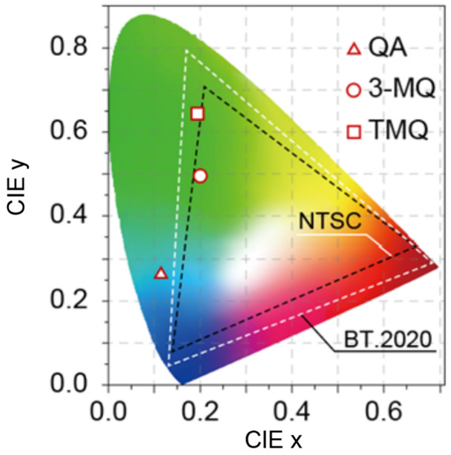

Supplementary Figure 40 | CIE coordinates diagram for RTP spectra of the QA, 3-MQ and TMQ in BP matrix under ambient conditions. Source data are provided as a Source Data file.

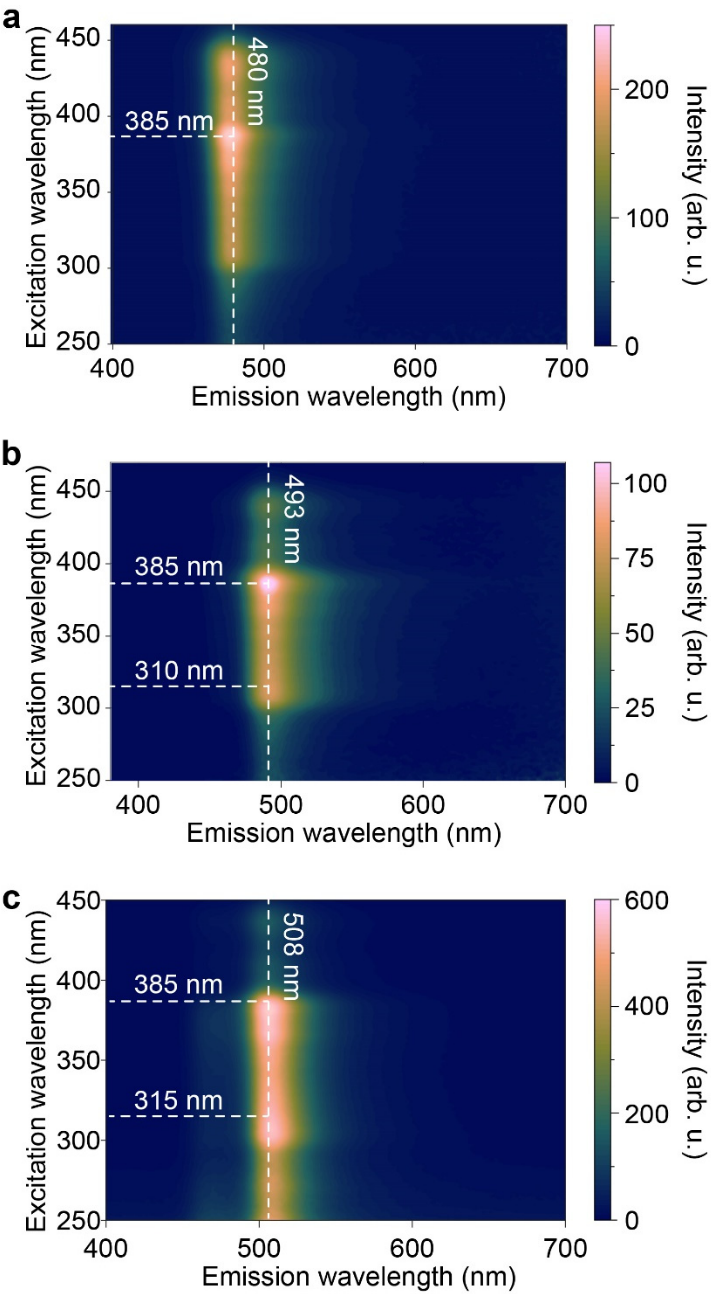

Supplementary Figure 41 | Excitation-phosphorescence mapping of QA (a), 3-MQ (b) and TMQ (c) phosphors in the BP matrix under ambient conditions. Source data are provided as a Source Data file.

Suppl. Note 6. Supporting date for the application of narrowband RTP

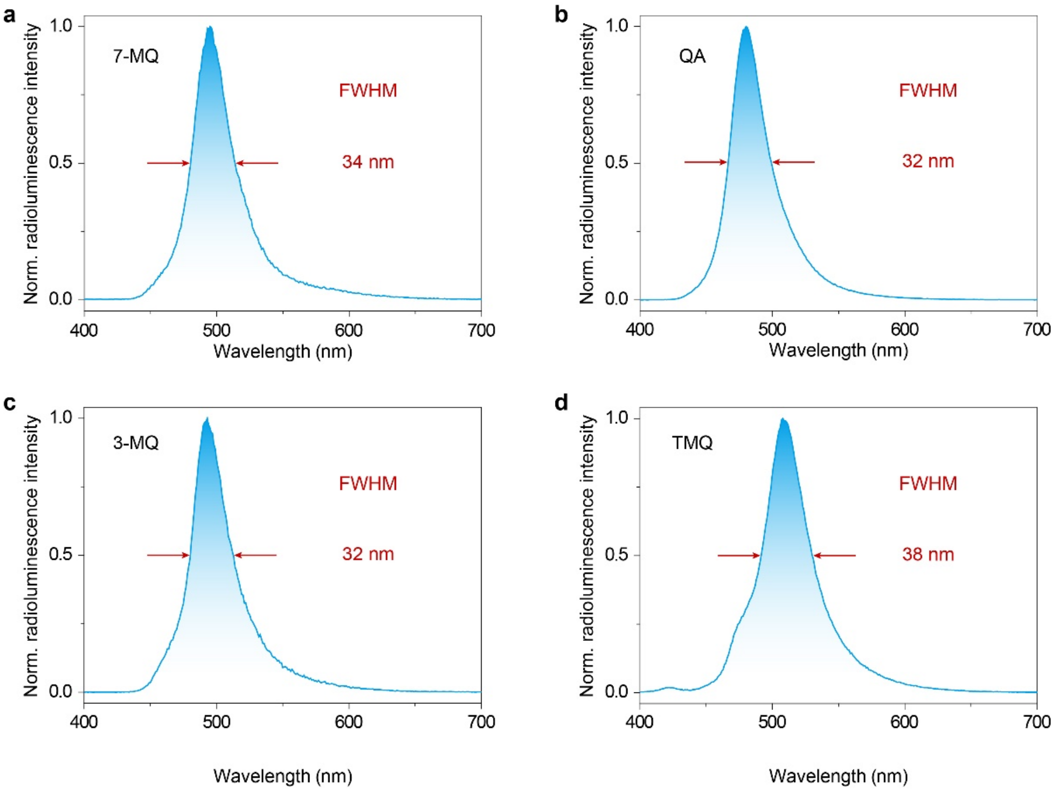

Supplementary Figure 42 | Normalized radioluminescence (RL) spectra of 7-MQ (a), QA (b), 3-MQ (c) and TMQ (d) in the BP matrix by X-ray. Source data are provided as a Source Data file.

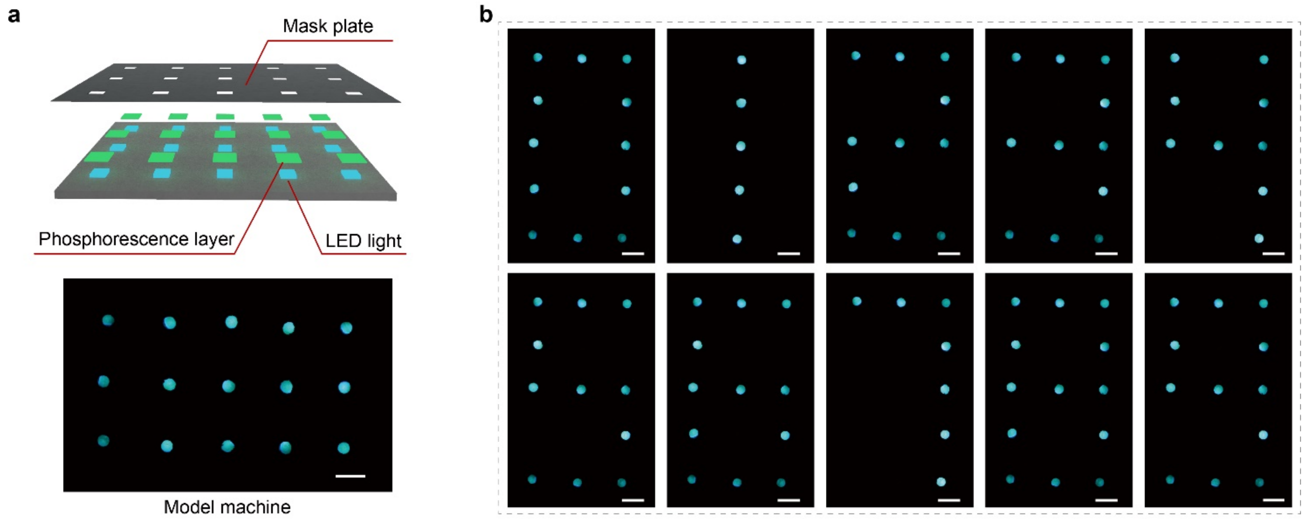

Supplementary Figure 43 | Schematic of the backlit display of the 7-MQ/BP materials. The scale bar is 1 cm.

Supplementary Reference

1. Su, Y. *et al.* Ultralong room temperature phosphorescence from amorphous organic materials toward confidential information encryption and decryption. *Sci. Adv.* **4**, eaas9732 (2018).
2. Tian, R., Xu, S. M., Xu, Q. & Lu, C. Large-scale preparation for efficient polymer-based room-temperature phosphorescence via click chemistry. *Sci. Adv.* **6** (2020).
3. Li, D., Yang, J., Fang, M. M., Tang, B. Z. & Li, Z. Stimulus-responsive room temperature phosphorescence materials with full-color tunability from pure organic amorphous polymers. *Sci. Adv.* **8**, eabl8392 (2022).
4. Peng, H. *et al.* On-demand modulating afterglow color of water-soluble polymers through phosphorescence FRET for multicolor security printing. *Sci. Adv.* **8** (2022).
5. Bolton, O., Lee, K., Kim, H. J., Lin, K. Y. & Kim, J. Activating efficient phosphorescence from purely organic materials by crystal design. *Nat. Chem.* **3**, 205-210 (2011).
6. Kwon, M. S. *et al.* Suppressing molecular motions for enhanced room-temperature phosphorescence of metal-free organic materials. *Nat. Commun.* **6** (2015).
7. An, Z. F. *et al.* Stabilizing triplet excited states for ultralong organic phosphorescence. *Nat. Mater.* **14**, 685-690 (2015).
8. Liu, Z. J. *et al.* Direct demonstration of triplet excimer in purely organic room temperature phosphorescence through rational molecular design. *Light-Sci Appl* **11** (2022).
9. He, Z. K. *et al.* White light emission from a single organic molecule with dual phosphorescence at room temperature. *Nat. Commun.* **8** (2017).
10. Li, Q. et al. Induction of long-lived room temperature phosphorescence of carbon dots by water in hydrogen-bonded matrices. Nat. Commun. **9**, 734 (2018).
11. Yang, J. et al. The influence of the molecular packing on the room temperature phosphorescence of purely organic luminogens. Nat. Commun. **9**, 840 (2018).
12. Wang, J. *et al.* A facile strategy for realizing room temperature phosphorescence and single molecule white light emission. *Nat. Commun.* **9**, 2963 (2018).
13. Zhao, W. *et al.* Boosting the efficiency of organic persistent room-temperature phosphorescence by intramolecular triplet-triplet energy transfer. *Nat. Commun.* **10**, 1595 (2019).
14. Cai, S. *et al.* Enabling long-lived organic room temperature phosphorescence in polymers by subunit interlocking. *Nat. Commun.* **10**, 4247 (2019).
15. Zhang, X. *et al.* Ultralong UV/mechano-excited room temperature phosphorescence from purely organic cluster excitons. *Nat. Commun.* **10**, 5161 (2019).
16. Gu, L. *et al.* Color-tunable ultralong organic room temperature phosphorescence from a multicomponent copolymer. *Nat. Commun.* **11**, 944 (2020).
17. Wu, X. *et al.* Exploiting racemism enhanced organic room-temperature phosphorescence to demonstrate Wallach's rule in the lighting chiral chromophores. *Nat. Commun.* **11**, 2145 (2020).
18. Feng, H. T. *et al.* Tuning molecular emission of organic emitters from fluorescence to phosphorescence through push-pull electronic effects. *Nat. Commun.* **11**, 2617 (2020).
19. Zhou, W. L. *et al.* Ultralong purely organic aqueous phosphorescence supramolecular polymer for targeted tumor cell imaging. *Nat. Commun.* **11**, 4655 (2020).
20. Xu, S. *et al.* Design of highly efficient deep-blue organic afterglow through guest sensitization and matrices rigidification. *Nat. Commun.* **11**, 4802 (2020).
21. Wang, T. *et al.* Thermochromic aggregation-induced dual phosphorescence via temperature-dependent sp(3)-

linked donor-acceptor electronic coupling. *Nat. Commun.* **12**, 1364 (2021).
22. Zhang, Y. *et al.* Ultraviolet irradiation-responsive dynamic ultralong organic phosphorescence in polymeric systems. *Nat. Commun.* **12**, 2297 (2021).
23. Xie, Z. *et al.* Wide-range lifetime-tunable and responsive ultralong organic phosphorescent multi-host/guest system. *Nat. Commun.* **12**, 3522 (2021).
24. Yang, J. *et al.* Rational design of pyrrole derivatives with aggregation-induced phosphorescence characteristics for time-resolved and two-photon luminescence imaging. *Nat. Commun.* **12**, 4883 (2021).
25. Xiao, F. *et al.* Guest-host doped strategy for constructing ultralong-lifetime near-infrared organic phosphorescence materials for bioimaging. *Nat. Commun.* **13**, 186 (2022).
26. Li, D. *et al.* Completely aqueous processable stimulus responsive organic room temperature phosphorescence materials with tunable afterglow color. *Nat. Commun.* **13**, 347 (2022).
27. Li, H. *et al.* Single-component color-tunable circularly polarized organic afterglow through chiral clusterization. *Nat. Commun.* **13**, 429 (2022).
28. Zhang, X. *et al.* Ultralong phosphorescence cellulose with excellent anti-bacterial, water-resistant and ease-to-process performance. *Nat. Commun.* **13**, 1117 (2022).
29. Zhu, T., Yang, T., Zhang, Q. & Yuan, W. Z. Clustering and halogen effects enabled red/near-infrared room temperature phosphorescence from aliphatic cyclic imides. *Nat. Commun.* **13**, 2658 (2022).
30. Liu, X. W. *et al.* Photo-thermo-induced room-temperature phosphorescence through solid-state molecular motion. *Nat. Commun.* **13**, 3887 (2022).
31. Yao, X. *et al.* Ultralong organic phosphorescence from isolated molecules with repulsive interactions for multifunctional applications. *Nat. Commun.* **13**, 4890 (2022).
32. Wan, K. *et al.* Structural materials with afterglow room temperature phosphorescence activated by lignin oxidation. *Nat. Commun.* **13**, 5508 (2022).
33. Huang, K. W. *et al.* Elastic organic crystals with ultralong phosphorescence for flexible anti-counterfeiting. *npj Flexible Electron.* **5** (2021).
34. Hamzehpoor, E. *et al.* Efficient room-temperature phosphorescence of covalent organic frameworks through covalent halogen doping. *Nat. Chem.* **15**, 83-90 (2023).
35. Ye, W. *et al.* Confining isolated chromophores for highly efficient blue phosphorescence. *Nat. Mater.* **20**, 1539-1544 (2021).
36. Gu, L. *et al.* Colour-tunable ultra-long organic phosphorescence of a single-component molecular crystal. *Nat. Photonics* **13**, 406-411 (2019).
37. Wang, X. *et al.* Organic phosphors with bright triplet excitons for efficient X-ray-excited luminescence. *Nat. Photonics* **15**, 187-192 (2021).
38. Chen, M. *et al.* Novel bipolar phenanthroimidazole derivative design for a nondoped deep-blue emitter with high singlet exciton yields. *Adv. Opt. Mater.* **3**, 1215-1219 (2015).
39. Li, C. Y. *et al.* Reversible luminescence switching of an organic solid: controllable on-off persistent room temperature phosphorescence and stimulated multiple fluorescence conversion. *Adv. Opt. Mater.* **3**, 1184-1190 (2015).
40. Yang, X. G. & Yan, D. P. Strongly enhanced long-lived persistent room temperature phosphorescence based on the formation of metal-organic hybrids. *Adv. Opt. Mater.* **4**, 897-905 (2016).
41. Chen, H., Yao, X. Y., Ma, X. & Tian, H. Amorphous, efficient, room-temperature phosphorescent metal-free polymers and their applications as encryption ink. *Adv. Opt. Mater.* **4**, 1397-1401 (2016).
42. Yu, L. *et al.* Pure organic emitter with simultaneous thermally activated delayed fluorescence and room-temperature phosphorescence: thermal-controlled triplet recycling channels. *Adv. Opt. Mater.* **5**, 1700588 (2017).

43. Li, M. P. *et al.* Prolonging ultralong organic phosphorescence lifetime to 2.5 s through confining rotation in molecular rotor. *Adv. Opt. Mater.* **7**, 1800820 (2019).
44. Chen, J. R. *et al.* Achieving dual-emissive and time-dependent evolutive organic afterglow by bridging molecules with weak intermolecular hydrogen bonding. *Adv. Opt. Mater.* **7**, 1801593 (2019).
45. Li, F. *et al.* Achieving dual persistent room‐temperature phosphorescence from polycyclic luminophores via inter-/intramolecular charge transfer. *Adv. Opt. Mater.* **7**, 1900511 (2019).
46. Li, J. J., Zhang, H. Y., Zhang, Y., Zhou, W. L. & Liu, Y. Room-temperature phosphorescence and reversible white light switch based on a cyclodextrin polypseudorotaxane xerogel. *Adv. Opt. Mater.* **7**, 1900589 (2019).
47. Feng, C. *et al.* Excited-state modulation for controlling fluorescence and phosphorescence pathways toward white-light emission. *Adv. Opt. Mater.* **7**, 1900767 (2019).
48. Ling, K. *et al.* Controllable multiemission with ultralong organic phosphorescence in crystal by isomerization. *Adv. Opt. Mater.* **7**, 1901076 (2019).
49. Wang, D. *et al.* Employing lactam copolymerization strategy to effectively achieve pure organic room‐temperature phosphorescence in amorphous state. *Adv. Opt. Mater.* **7**, 1901277 (2019).
50. Choi, K. H., Lee, K. H., Lee, J. Y. & Kim, T. Simultaneous achievement of high efficiency and long lifetime in deep blue phosphorescent organic light-emitting diodes. *Adv. Opt. Mater.* **7**, 1901374 (2019).
51. She, P. F. *et al.* Controlling organic room temperature phosphorescence through external heavy-atom effect for white light emission and luminescence printing. *Adv. Opt. Mater.* **8**, 1901437 (2020).
52. Wen, Y. T. *et al.* Achieving highly efficient pure organic single-molecule white-light emitter: the coenhanced fluorescence and phosphorescence dual emission by tailoring alkoxy substituents. *Adv. Opt. Mater.* **8**, 1901995 (2020).
53. Liu, B. T., Liu, E. P., Sa, R. J. & Liu, T. F. Crystalline hydrogen‐bonded organic chains achieving ultralong phosphorescence via triplet–triplet energy transfer. *Adv. Opt. Mater.* **8**, 2000281 (2020).
54. Louis, M. *et al.* Biluminescence under ambient conditions: water‐soluble organic emitter in high-oxygen-barrier polymer. *Adv. Opt. Mater.* **8**, 2000427 (2020).
55. Fang, W. J. *et al.* Versatile induction of efficient organic-based room-temperature phosphorescence via al-DMSO matrices encapsulation. *Adv. Opt. Mater.* **8**, 2000482 (2020).
56. Wu, B. *et al.* Ultralong and high-efficiency room temperature phosphorescence of organic-phosphors-doped polymer films enhanced by 3D network. *Adv. Opt. Mater.* **8**, 2001192 (2020).
57. Ruan, Z. J. *et al.* Luminous butterflies: rational molecular design to optimize crystal packing for dramatically enhanced room-temperature phosphorescence. *Adv. Opt. Mater.* **9**, 2001549 (2021).
58. Chen, J. R. *et al.* A multi-stimuli-responsive molecule with responses to light, oxygen, and mechanical stress through flexible tuning of triplet excitons. *Adv. Opt. Mater.* **9**, 2001550 (2021).
59. Wang, Z. *et al.* Optical waveguide color tuning by fluorescence-phosphorescence dual emission and disparity of optical losses. *Adv. Opt. Mater.* **9**, 2001591 (2021).
60. Li, X. S., Wu, Y., Zhao, Y. R. & Yu, Z. Q. Employing cholesterol copolymerization strategy for a thermally processable organic room-temperature phosphorescence material. *Adv. Opt. Mater.* **9**, 2001893 (2021).
61. Sun, Y., Wang, G., Li, X., Zhou, B. & Zhang, K. Achieving high afterglow brightness in organic dopant‐matrix systems. *Adv. Opt. Mater.* **9**, 2100353 (2021).
62. Zhou, Z. *et al.* Energy transfer mediated enhancement of room-temperature phosphorescence of carbon dots embedded in matrixes. *Adv. Opt. Mater.* **10**, 2100704 (2021).
63. Zhu, Y. *et al.* Ultralong polymeric room temperature phosphorescence materials fabricated by multiple hydrogen bondings resistant to temperature and humidity. *Adv. Opt. Mater.* **9**, 2100782 (2021).
64. Zhou, Y. *et al.* Deep-blue ultralong room-temperature phosphorescence from halogen-free organic materials through cage effect for various applications. *Adv. Opt. Mater.* **9**, 2100959 (2021).

65. Wang, Y. *et al.* Persistent organic white-emitting afterglow from ultralong thermally activated delayed fluorescence and room-temperature phosphorescence. *Adv. Opt. Mater.* **9**, 2101075 (2021).
66. Wu, B., Xu, X., Tang, Y., Han, X. & Wang, G. Multifunctional optical polymeric films with photochromic, fluorescent, and ultra-long room temperature phosphorescent properties. *Adv. Opt. Mater.* **9**, 2101266 (2021).
67. Gao, L. *et al.* Water-induced blue-green variable nonconventional ultralong room temperature phosphorescence from cross-linked copolymers *via* click chemistry. *Adv. Opt. Mater.* **9**, 2101284 (2021).
68. Huang, G. *et al.* Long-range charge transportation induced organic host-guest dual color long persistent luminescence. *Adv. Opt. Mater.* **9**, 2101337 (2021).
69. Lin, X., Xu, Q. & Ma, X. Emission-tunable room-temperature phosphorescent polymers based on dynamic reversible supramolecule-mediated photocrosslinking. *Adv. Opt. Mater.* **10**, 2101646 (2021).
70. Li, F. Y. *et al.* Color-tunable dual persistent emission via a triplet exciton reservoir for temperature sensing and anti-counterfeiting. *Adv. Opt. Mater.* **10**, 2101773 (2022).
71. Sun, Y. *et al.* Manipulation of triplet excited states for long-lived and efficient organic afterglow. *Adv. Opt. Mater.* **10**, 2101909 (2021).
72. Zhang, D. *et al.* On-demand circularly polarized room-temperature phosphorescence in chiral nematic nanoporous silica films. *Adv. Opt. Mater.* **10**, 2102015 (2022).
73. Zhang, Y. *et al.* Photo-controlled reversible multicolor room-temperature phosphorescent solid supramolecular pseudopolyrotaxane. *Adv. Opt. Mater.* **10**, 2102169 (2022).
74. Wu, Y. Y. *et al.* Confining carboxylized carbon nanotube for phosphorescence afterglow with optical memory plasticity. *Adv. Opt. Mater.* **10**, 2102323 (2022).
75. Tao, W. *et al.* Strain-responsive persistent room-temperature phosphorescence from halogen-free polymers for early damage reporting through phosphorescence lifetime and image analysis. *Adv. Opt. Mater.* **10**, 2102449 (2022).
76. Zhao, Y. *et al.* Visible light activated organic room-temperature phosphorescence based on triplet-to-singlet Förster-resonance energy transfer. *Adv. Opt. Mater.* **10**, 2102701 (2022).
77. She, P. F. *et al.* Single-component molecular dual persistent room temperature phosphorescence from low- and high-lying triplet states. *Adv. Opt. Mater.* **10**, 2102706 (2022).
78. Liu, H. *et al.* Dual‐Emission of fluorescence and room-temperature phosphorescence for ratiometric and colorimetric oxygen sensing and detection based on dispersion of pure organic thianthrene dimer in polymer host. *Adv. Opt. Mater.* **10**, 2102814 (2022).
79. Yuan, S. *et al.* Effectively unlocking the potential molecular room temperature phosphorescence of pure carbazole derivatives. *Adv. Opt. Mater.* **10**, 2200090 (2022).
80. Nie, X. *et al.* Broad-band visible-light excitable room-temperature phosphorescence via polymer site-isolated dye aggregates. *Adv. Opt. Mater.* **10**, 2200099 (2022).
81. Shen, F. F. *et al.* Macrocyclic confined purely organic room‐temperature phosphorescence three-photon targeted imaging. *Adv. Opt. Mater.* **10**, 2200245 (2022).
82. Xu, X. & Yan, B. Base-tuning HOF-based host-guest ultralong organic phosphorescence systems with phosphorescent thermochromism using for information security and thermometer. *Adv. Opt. Mater.* **10**, 2200451 (2022).
83. Wang, Z. *et al.* Regulation of irradiatio-dependent long-lived room temperature phosphorescence by controlling molecular structures of chromophores and matrix. *Adv. Opt. Mater.* **10**, 2200481 (2022).
84. Wang, D. H. *et al.* Boric acid-activated room-temperature phosphorescence and thermally activated delayed fluorescence for efficient solid-state photoluminescence materials. *Adv. Opt. Mater.* **10**, 2200629 (2022).
85. Xie, N. *et al.* A benzene ring-linked dimethylamino and borate ester-based molecule and organic crystal: efficient dual room-temperature phosphorescence with responsive property. *Adv. Opt. Mater.* **10**, 2200767

(2022).
86. Wang, J. M. *et al.* Organic hollow microstructures with room temperature phosphorescence. *Adv. Opt. Mater.* **10**, 2200985 (2022).
87. Liang, D. *et al.* Room-temperature phosphorescence enhanced by narrowing down ΔE_{st} through tuning excited states energy levels. *Adv. Opt. Mater.* **10**, 2201130 (2022).
88. Garain, S. *et al.* Arylene diimide phosphors: aggregation modulated twin room temperature phosphorescence from pyromellitic diimides. *Angew. Chem. Int. Ed.* **60**, 12323-12327 (2021).
89. Zheng, H. Y., Cao, P. S., Wang, Y. Y., Lu, X. M. & Wu, P. Ultralong room-temperature phosphorescence from boric acid. *Angew. Chem. Int. Ed.* **60**, 9500-9506 (2021).
90. Ren, J. *et al.* Force-induced turn-on persistent room-temperature phosphorescence in purely organic luminogen. *Angew. Chem. Int. Ed.* **60**, 12335-12340 (2021).
91. Zhang, J. Y. *et al.* Stimuli-responsive deep-blue organic ultralong phosphorescence with lifetime over 5 s for reversible water-jet anti-counterfeiting printing. *Angew. Chem. Int. Ed.* **60**, 17094-17101 (2021).
92. Chen, B. A. *et al.* An Organic host-guest system producing room-temperature phosphorescence at the parts-per-billion level. *Angew. Chem. Int. Ed.* **60**, 16970-16973 (2021).
93. Sun, S. Y., Wang, J., Ma, L. W., Ma, X. & Tian, H. A universal strategy for organic fluid phosphorescence materials. *Angew. Chem. Int. Ed.* **60**, 18557-18560 (2021).
94. Tian, Y. *et al.* Multistage stimulus-responsive room temperature phosphorescence based on host-guest doping systems. *Angew. Chem. Int. Ed.* **60**, 20259-20263 (2021).
95. H., Sun, S. Y., Ma, X. & Tian, H. Activating room-temperature phosphorescence of organic luminophores via external heavy-atom effect and rigidity of ionic polymer matrix. *Angew. Chem. Int. Ed.* **60**, 19735-19739 (2021).
96. Dong, C. M. *et al.* Influence of isomerism on radioluminescence of purely organic phosphorescence scintillators. *Angew. Chem. Int. Ed.* **60**, 27195-27200 (2021).
97. Li, G. F. *et al.* Organic supramolecular zippers with ultralong organic phosphorescence by a Dexter energy transfer mechanism. *Angew. Chem. Int. Ed.* **61**, e202113425 (2022).
98. Huo, M., Dai, X. Y. & Liu, Y. Ultrahigh supramolecular cascaded room-temperature phosphorescence capturing system. *Angew. Chem. Int. Ed.* **60**, 27171-27177 (2021).
99. Wang, X. H. *et al.* Reversible photoswitching between fluorescence and room temperature phosphorescence by manipulating excited state dynamics in molecular aggregates. *Angew. Chem. Int. Ed.* **61**, e202114264 (2022).
100. Xu, W. W. *et al.* Tunable second-level room-temperature phosphorescence of solid supramolecules between acrylamide-phenylpyridium copolymers and cucurbit[7]uril. *Angew. Chem. Int. Ed.* **61**, e202115265 (2022).
101. Garain, S., Sarkar, S., Garain, B. C., Pati, S. K. & George, S. J. Chiral arylene diimide phosphors: circularly polarized ambient phosphorescence from bischromophoric pyromellitic diimides. *Angew. Chem. Int. Ed.* **61**, e202115773 (2022).
102. Dai, W. B. *et al.* Halogen bonding: A new platform for achieving multi-stimuli-responsive persistent phosphorescence. *Angew. Chem. Int. Ed.* **61**, e202200236 (2022).
103. Wan, K. L. *et al.* Sustainable afterglow room-temperature phosphorescence emission materials generated using natural phenolics. *Angew. Chem. Int. Ed.* **61**, e202202760 (2022).
104. Huang, W. B., Fu, C. Y., Liang, Z. W., Zhou, K. & He, Z. K. Strong circularly-polarized room-temperature phosphorescence from a feasibly separable scaffold of bidibenzo[b,d]furan with locked axial chirality. *Angew. Chem. Int. Ed.* **61**, e202202977 (2022).
105. Song, J., Ma, L., Sun, S., Tian, H. & Ma, X. Reversible multilevel stimuli-responsiveness and multicolor room-temperature phosphorescence emission based on a single-component system. *Angew. Chem. Int. Ed.* **61**,

e202206157 (2022).
106. Zheng, x. *et al.* nearly unity quantum yield persistent room-temperature phosphorescence from heavy atom-free rigid inorganic/organic hybrid frameworks. *Angew. Chem. Int. Ed.* **61**, e202209343 (2022).
107. Li, M. *et al.* Molecular engineering of sulfur-bridged polycyclic emitters towards tunable tadf and rtp electroluminescence. *Angew. Chem. Int. Ed.* **61**, e202209343 (2022).
108. Wang, H. J., Xing, W. W., Zhang, H. Y., Xu, W. W. & Liu, Y. Cucurbit[8]uril confined 6-bromoisoquinoline derivative dicationic phosphorescent energy transfer supramolecular switch for lysosome targeted imaging. *Adv. Opt. Mater.* **10**, 2201178 (2022).
109. Sun, Y. H. *et al.* Purely organic blue room-temperature phosphorescence activated by acrylamide in situ photopolymerization. *Adv. Opt. Mater.* **10**, 2201330 (2022).
110. Liu, J. H. *et al.* Organic afterglow emulsions exhibiting 2.4 s phosphorescence lifetimes and specific protein binding property. *Adv. Opt. Mater.* **10**, 2201502 (2022).
111. Sun, Z. B. *et al.* Shape-memorable, self-healable, recyclable, and full-color emissive ultralong organic phosphorescence vitrimers with exchangeable covalent bonds. *Adv. Opt. Mater.* **10**, 2201558 (2022).
112. Liu, M. *et al.* Kilogram-scale fabricated organic long-persistent luminescence materials with multi-level temperature response. *Adv. Opt. Mater.* **10**, 2201684 (2022).
113. Yu, J., Wang, H. & Liu, Y. Double-network confined supramolecular phosphorescence light-harvesting boosting photocatalysis. *Adv. Opt. Mater.* **10**, 2201761 (2022).
114. Wang, H. J. *et al.* Noncovalent bridged bis(coumarin-24-crown-8) phosphorescent supramolecular switch. *Adv. Opt. Mater.* **10**, 2201903 (2022).
115. Cho, Y. J., Jeon, S. K., Chin, B. D., Yu, E. & Lee, J. Y. The design of dual emitting cores for green thermally activated delayed fluorescent materials. *Angew. Chem. Int. Ed.* **54**, 5201-5204 (2015).
116. Wang, S. *et al.* Highly efficient near-infrared delayed fluorescence organic light emitting diodes using a phenanthrene-based charge-transfer compound. *Angew. Chem. Int. Ed.* **54**, 13068-13072 (2015).
117. Yang, Z. *et al.* Intermolecular Electronic Coupling of Organic Units for Efficient Persistent Room-Temperature Phosphorescence. *Angew. Chem. Int. Ed.* **55**, 2181-2185 (2016).
118. Yin, Z. *et al.* Molecular engineering through control of structural deformation for highly efficient ultralong organic phosphorescence. *Angew. Chem. Int. Ed.* **60**, 2058-2063 (2021).
119. Liu, W. X. *et al.* Room-temperature phosphorescence invoked through norbornyl-driven intermolecular interaction intensification with anomalous reversible solid-state photochromism. *Angew. Chem. Int. Ed.* **59**, 20161-20166 (2020).
120. Liao, Q. Y. *et al.* 9,9-Dimethylxanthene derivatives with room-temperature phosphorescence: substituent effects and emissive properties. *Angew. Chem. Int. Ed.* **59**, 9946-9951 (2020).
121. Wang, J., Huang, Z. Z., Ma, X. & Tian, H. Visible-light-excited room-temperature phosphorescence in water by cucurbit[8]uril-mediated supramolecular assembly. *Angew. Chem. Int. Ed.***59**, 9928-9933 (2020).
122. Wang, H. *et al.* Amorphous ionic polymers with color-tunable ultralong organic phosphorescence. *Angew. Chem. Int. Ed.* **58**, 18776-18782 (2019).
123. Wang, J. Q. *et al.* Bromine-substituted fluorene: molecular structure, Br-Br interactions, room-temperature phosphorescence, and tricolor triboluminescence. *Angew. Chem. Int. Ed.* **57**, 16821-16826 (2018).
124. Gu, L. *et al.* Dynamic ultralong organic phosphorescence by photoactivation. *Angew. Chem. Int. Ed.* **57**, 8425-8431 (2018).
125. Lin, X. H., Wang, J., Ding, B. B., Ma, X. & Tian, H. Tunable-emission amorphous room-temperature phosphorescent polymers based on thermoreversible dynamic covalent bonds. *Angew. Chem. Int. Ed.* **60**, 3459-3463 (2021).
126. Demangeat, C. *et al.* σ-Conjugation and H-Bond-directed supramolecular self-assembly: Key features for

efficient long-lived room temperature phosphorescent organic molecular crystals. *Angew. Chem. Int. Ed.* **60**, 2446-2454 (2021).

127. Zhang, Z. Y. *et al.* A synergistic enhancement strategy for realizing ultralong and efficient room-temperature phosphorescence. *Angew. Chem. Int. Ed.* **59**, 18748-18754 (2020).
128. Lei, Y. X. *et al.* Wide-Range Color-tunable organic phosphorescence materials for printable and writable security inks. *Angew. Chem. Int. Ed.* **59**, 16054-16060 (2020).
129. Li, H. *et al.* Stimuli-responsive circularly polarized organic ultralong room temperature phosphorescence. *Angew. Chem. Int. Ed.* **59**, 4756-4762 (2020).
130. Liang, X. *et al.* Organic room-temperature phosphorescence with strong circularly polarized luminescence based on paracyclophanes. *Angew. Chem. Int. Ed.* **58**, 17220-17225 (2019).
131. Xu, W. *et al.* Self-stabilized amorphous organic materials with room-temperature phosphorescence. *Angew. Chem. Int. Ed.* **58**, 16018-16027 (2019).
132. Zhang, Z. Y., Chen, Y. & Liu, Y. Efficient room-temperature phosphorescence of a solid-state supramolecule enhanced by cucurbit[6]uril. *Angew. Chem. Int. Ed.* **58**, 6028-6032 (2019).
133. Xiong, Y. *et al.* Designing efficient and ultralong pure organic room-temperature phosphorescent materials by structural isomerism. *Angew. Chem. Int. Ed.* **57**, 7997-8001 (2018).
134. Chen, X. F. *et al.* Versatile room-temperature-phosphorescent materials prepared from *N*-substituted naphthalimides: emission enhancement and chemical conjugation. *Angew. Chem. Int. Ed.* **55**, 9872-9876 (2016).
135. Wei, J. B. *et al.* Induction of strong long-lived room-temperature phosphorescence of *N*-phenyl-2-naphthylamine molecules by confinement in a crystalline dibromobiphenyl matrix. *Angew. Chem. Int. Ed.* **55**, 15589-15593 (2016).
136. Yang, J. *et al.* Elucidating the excited state of mechanoluminescence in organic luminogens with room-temperature phosphorescence. *Angew. Chem. Int. Ed.* **56**, 15299-15303 (2017).
137. Yu, Y. C. *et al.* Room-temperature-phosphorescence-based dissolved oxygen detection by core-shell polymer nanoparticles containing metal-free organic phosphors. *Angew. Chem. Int. Ed.* **56**, 16207-16211 (2017).
138. Cheng, Z. C. *et al.* Ultralong phosphorescence from organic ionic crystals under ambient conditions. *Angew. Chem. Int. Ed.* **57**, 678-682 (2018).
139. Xie, Z. L. *et al.* White-light emission strategy of a single organic compound with aggregation-induced emission and delayed fluorescence properties. *Angew. Chem. Int. Ed.* **54**, 7181-7184 (2015).
140. Ma, L. W., Sun, S. Y., Ding, B. B., Ma, X. & Tian, H. Highly efficient room-temperature phosphorescence based on single-benzene structure molecules and photoactivated luminescence with afterglow. *Adv. Funct. Mater.* **31** (2021).
141. Kuila, S., Garain, S., Bandi, S. & George, S. J. All-organic, temporally pure white afterglow in amorphous films using complementary blue and greenish-yellow ultralong room temperature phosphors. *Adv. Funct. Mater.* **30**, 2003693 (2020).
142. Wang, X. F. *et al.* Pure organic room temperature phosphorescence from unique micelle-assisted assembly of nanocrystals in water. *Adv. Funct. Mater.* **30**, 1907282 (2020).
143. Zhou, C. J. *et al.* Ternary emission of fluorescence and dual phosphorescence at room temperature: a single-molecule white light emitter based on pure organic aza-aromatic material. *Adv. Funct. Mater.* **28**, 1802407 (2018).
144. Ogoshi, T. *et al.* Ultralong room-temperature phosphorescence from amorphous polymer poly(styrene sulfonic acid) in air in the dry solid state. *Adv. Funct. Mater.* **28**, 1707369 (2018).
145. Cai, S. Z. *et al.* Enhancing ultralong organic phosphorescence by effective π-type halogen bonding. *Adv. Funct. Mater.* **28**, 1705045 (2018).

146. Lee, D. *et al.* Room temperature phosphorescence of metal-free organic materials in amorphous polymer matrices. *J. Am. Chem. Soc.* **135**, 6325-6329 (2013).
147. Wang, X. F. *et al.* Pure organic room temperature phosphorescence from excited dimers in self-assembled nanoparticles under visible and near-infrared irradiation in water. *J. Am. Chem. Soc.* **141**, 5045-5050 (2019).
148. Gu, L. *et al.* Circularly polarized organic room temperature phosphorescence from amorphous copolymers. *J. Am. Chem. Soc.* **143**, 18527-18535 (2021).
149. Zhang, Y. *et al.* Large-area, flexible, transparent, and long-lived polymer-based phosphorescence films. *J. Am. Chem. Soc.* **143**, 13675-13685 (2021).
150. Yu, H. J. *et al.* Photooxidation-driven purely organic room-temperature phosphorescent lysosome-targeted imaging. *J. Am. Chem. Soc.* **143**, 13887-13894 (2021).
151. Cai, S. *et al.* Ultralong organic phosphorescent foams with high mechanical strength. *J. Am. Chem. Soc.* **143**, 16256-16263 (2021).
152. Tao, Y. *et al.* Resonance-induced stimuli-responsive capacity modulation of organic ultralong room temperature phosphorescence. *J. Am. Chem. Soc.* **144**, 6946-6953 (2022).
153. Zhang, Y. *et al.* Cross-linked polyphosphazene nanospheres boosting long-lived organic room-temperature phosphorescence. *J. Am. Chem. Soc.* **144**, 6107-6117 (2022).
154. Garain, S. *et al.* Anion-π-Induced room temperature phosphorescence from emissive charge-transfer states. *J. Am. Chem. Soc.* **144**, 10854-10861 (2022).
155. Shoji, Y. *et al.* Unveiling a new aspect of simple arylboronic esters: long-lived room-temperature phosphorescence from heavy-atom-free molecules. *J. Am. Chem. Soc.* **139**, 2728-2733 (2017).
156. Li, D. *et al.* Amorphous metal-free room-temperature phosphorescent small molecules with multicolor photoluminescence via a host–guest and dual-emission strategy. *J. Am. Chem. Soc.* **140**, 1916-1923 (2018).
157. Bian, L. *et al.* Simultaneously enhancing efficiency and lifetime of ultralong organic phosphorescence materials by molecular self-assembly. *J. Am. Chem. Soc.* **140**, 10734-10739 (2018).
158. Ren, Y. *et al.* Clusterization-triggered color-tunable room-temperature phosphorescence from 1,4-dihydropyridine-based polymers. *J. Am. Chem. Soc.* **144**, 1361-1369 (2022).
159. Liu, Z. Y. *et al.* Sulfur-based intramolecular hydrogen-bond: excited-state hydrogen-bond on/off switch with dual room-temperature phosphorescence. *J. Am. Chem. Soc.* **141**, 9885-9894 (2019).
160. Shi, Y. *et al.* Red phosphorescent carbon quantum dot organic framework-based electroluminescent light-emitting diodes exceeding 5% external quantum efficiency. *J. Am. Chem. Soc.* **143**, 18941-18951 (2021).
161. Cui, X. Y., Shi, W. Y. & Lu, C. Control of multicolor and white emission by triplet energy transfer. *J. Phys. Chem. A* **125**, 4209-4215 (2021).
162. Sun, W. *et al.* Protonation-induced room-temperature phosphorescence in fluorescent polyurethane. *J. Phys. Chem. A* **121**, 4225-4232 (2017).
163. Raj, A. M., Sharma, G., Prabhakar, R. & Ramamurthy, V. Room-temperature phosphorescence from encapsulated pyrene induced by xenon. *J. Phys. Chem. A* **123**, 9123-9131 (2019).
164. Chen, X., Liu, Z. F. & Jin, W. J. The effect of electron donation and intermolecular interactions on ultralong phosphorescence lifetime of 4-carnoyl phenylboronic acids. *J. Phys. Chem. A* **124**, 2746-2754 (2020).
165. Yuan, J. *et al.* Activating intersystem crossing and aggregation coupling by cn-substitution for efficient organic ultralong room temperature phosphorescence. *J. Phys. Chem. C* **124**, 10129-10134 (2020).
166. Shimizu, M. *et al.* Siloxy group-induced highly efficient room temperature phosphorescence with long lifetime. *J. Phys. Chem. C* **120**, 11631-11639 (2016).
167. Bhattacharjee, I., Acharya, N., Karmakar, S. & Ray, D. Room-temperature orange-red phosphorescence by way of intermolecular charge transfer in single-component phenoxazine-quinoline conjugates and chemical sensing. *J. Phys. Chem. C* **122**, 21589-21597 (2018).

168. DeRosa, C. A., Hiroto, S. & Fraser, C. L. Amplified heavy-atom free phosphorescence from meta-dimethoxy difluoroboron *β*-diketonate charge-transfer materials. *J. Phys. Chem. C* **123**, 20488-20496 (2019).
169. Wu, H. *et al.* Molecular phosphorescence in polymer matrix with reversible sensitivity. *ACS Appl. Mater. Interfaces* **12**, 20765-20774 (2020).
170. Wu, H. W. *et al.* Multidimensional structure conformation of persulfurated benzene for highly efficient phosphorescence. *ACS Appl. Mater. Interfaces* **13**, 1314-1322 (2021).
171. Higginbotham, H. F. *et al.* Heavy-atom-free room-temperature phosphorescent organic light-emitting diodes enabled by excited states engineering. *ACS Appl. Mater. Interfaces* **13**, 2899-2907 (2021).
172. Xu, W. *et al.* Supramolecule-originated emission: A room-temperature phosphorescence 2D ionic H-Bond network from nonemissive aliphatic derivatives. *ACS Appl. Mater. Interfaces* **13**, 61528-61535 (2021).
173. Zhang, X. *et al.* Irreversible humidity-responsive phosphorescence materials from cellulose for advanced anti-counterfeiting and environmental monitoring. *ACS Appl. Mater. Interfaces* **14**, 16582-16591(2022).
174. Zheng, Y. *et al.* Long-lived room temperature phosphorescence crystals with green light excitation. *ACS Appl. Mater. Interfaces* **14**, 15706-15715 (2022).
175. Wang, L., Hao, A. Y. & Xing, P. Y. Steroid-aromatics clathrates as chiroptical materials with circularly polarized luminescence and phosphorescence. *ACS Appl. Mater. Interfaces* **14**, 44902-44908, (2022).
176. Zhou, C. *et al.* Waterborne polyurethanes with tunable fluorescence and room-temperature phosphorescence. *ACS Appl. Mater. Interfaces* **7**, 17209-17216(2015).
177. Deng, Y. C., Li, P., Li, J. T., Sun, D. L. & Li, H. R. Color-tunable aqueous room-temperature phosphorescence supramolecular assembly. *ACS Appl. Mater. Interfaces* **13**, 14420-14429 (2021).
178. Katsurada, Y., Hirata, S., Totani, K., Watanabe, T. & Vacha, M. Photoreversible on–off recording of persistent room-temperature phosphorescence. *Adv. Opt. Mater.* **3**, 1726-1737(2015).
179. Kang, D. H., Zeng, Y. Y., Tewari, M. & Kim, J. Highly sensitive and quantitative biodetection with lipid-polymer hybrid nanoparticles having organic room-temperature phosphorescence. *Biosens. Bioelectron.* **199**, 113889 (2022).
180. Lee, D. R., Park, J. & Lee, J. Y. Metal and halogen-free purely organic room temperature phosphorescence material using heavy atom effect of phenoselenazine. *Org. Electron*. **106**, 106534 (2022).
181. Yue, L. T. *et al.* Organic phosphor doped thermoplastics with ultralong and memorable room temperature phosphorescence different from crystals. *Chem. Eng. J* **433**, 134307 (2022).
182. Deng, L. S. *et al.* Regulating excited state of sulfone-locked triphenylamine heteroaromatics for high-efficiency ultralong room-temperature phosphorescence. *Chem. Eng. J* **449**, 137834 (2022).
183. Skuodis, E. *et al.* Very sensitive probes for quantitative and organoleptic detection of oxygen based on conformer-induced room-temperature phosphorescence enhancement of the derivative of triazatruxene and phenothiazine. *Sens. Actuators B Chem.* **373**, 132727 (2022).
184. Xu, Y. H. *et al.* Efficient ultralong and color-tunable room-temperature phosphorescence from polyacrylamide platform by introducing sulfanilic acid. *Chem. Eng. J* **453**, 139753 (2023).
185. Huang, Y. S. *et al.* Multimode stimuli responsive dual-state organic room temperature phosphorescence from a phenanthrene derivative. *Chem. Eng. J* **444**, 136629 (2022).
186. Nie, F., Zhou, B. & Yan, D. P. Ultralong room temperature phosphorescence and reversible mechanochromic luminescence in ionic crystals with structural isomerism. *Chem. Eng. J* **453**, 139806 (2023).
187. Zhang, Y. F. *et al.* In-situ grafting *N*-arylcarbazoles enables more ultra-long room temperature phosphorescence polymers. *Chem. Eng. J* **452**, 139385 (2023).
188. Ni, Y. Y. *et al.* Room-temperature phosphorescence based on chitosan carbon dots for trace water detection in organic solvents and anti-counterfeiting application. *Dyes Pigm.* **197**, 109923 (2022).
189. Zhang, X. P. *et al.* Highly efficient and persistent room temperature phosphorescence from cluster exciton

enables ultrasensitive off-on voc sensing. *Matter* **5**, 3499-3512(2022).

190. Li, Z. T., Ding, B. B., Liu, X. Q., Sun, L. B. & Ma, X. Se/S enhanced room-temperature phosphorescence of organic polymers. *Dyes Pigm.* **195**, 109663 (2021).
191. Zhou, W. *et al.* Room temperature phosphorescence of heavy-atom-free indole carboxylic acid/polyacrylamide: Low cost, long lifetime and good luminescent efficiency. *Dyes Pigm.* **205**, 110481 (2022).
192. He, P. Y. *et al.* Constructing matrix-free solid-state carbon dots with efficient room-temperature phosphorescence. *J. Lumin.* **253**, 119454 (2023).
193. She, P. *et al.* Controllable photoactivated organic persistent room-temperature phosphorescence for information encryption and visual temperature detection. *Cell Rep. Physic. Sci.* **2**, 100505 (2021).
194. Zhang, Y. F. *et al.* Evoking ultra-long molecular room temperature phosphorescence of pure carbazole derivatives. *Chem. Eng. J* **447**, 137458 (2022).
195. Han, S. H. *et al.* Achieving long lifetime of pure organic room-temperature phosphorescence via constructing hydrogen-bonded organic frameworks. *J. Lumin.* **236**, 118120 (2021).
196. Zhang, Z. *et al.* Highly efficient and stable deep-blue room temperature phosphorescence via through-space conjugation. *Chem. Eng. J* **442**, 136179 (2022).
197. Tian, Y. *et al.* Adjusting organic room-temperature phosphorescence with orderly stimulus-responsive molecular motion in crystals. *Cell Rep. Physic. Sci.* **1**, 100052 (2020).
198. Gao, Q. *et al.* Stereospecific redox-mediated clusterization reconstruction for constructing long-lived, color-tunable, and processable phosphorescence cellulose. *Chem. Eng. J* **451**, 138923 (2023).
199. Song, Z. M., Lee, J. & Zhu, H. J. Multi-stimuli-responsive aryl-sulfone derivatives with room-temperature phosphorescence and mechanoluminescence properties. *J. Lumin.* **251**, 119223 (2022).
200. Zheng, K. L. *et al.* Multicolor ultralong room-temperature phosphorescence from pure organic emitters by structural isomerism. *Chem. Eng. J* **408**, 127309 (2021).
201. Chen, X. Q. *et al.* Fluorene-based host-guest phosphorescence materials for information encryption. *Chem. Eng. J* **426**, 131607 (2021).
202. Yuan, J. *et al.* Sustainable afterglow materials from lignin inspired by wood phosphorescence. *Cell Rep. Physic. Sci.* **2**, 100542 (2021).
203. Zan, M. H. *et al.* A facile strategy to realize metal-free room-temperature phosphorescence by construct nitrogen doped carbon dots-based nanocomposite. *Microchem. J.* **172**, 106878 (2022).
204. Zhou, M.-S. *et al.* Multiemission tunability with ultralong and time–dependent room-temperature phosphorescence from isophthalic acid-decorated carbazole by coordination–induced crystallization. *Dyes Pigm.* **195**, 109715 (2021).
205. Garain, S., Singh, A. K., Peter, S. C. & George, S. J. Ambient, tunable room temperature phosphorescence from a simple phthalimide phosphor in amorphous polymeric matrix and in crystalline state. *Mater. Res. Bull.* **142**, 111420 (2021).
206. Kong, Y. L., Lei, T. T., He, Y. & Song, G. W. Background-free room temperature phosphorescence and digital image colorimetry detection of melamine by carbon nitride quantum dots in cellulose matrix with smartphone-based portable device. *Food Chem.* **390**, 133135 (2022).
207. Goudappagouda, Nidhankar, A. D., Nayak, R. A. & Babu, S. S. Aggregation-induced phosphorescence of an anthraquinone based emitter. *Org. Biomol. Chem.* **19**, 1004-1008 (2021).
208. Han, S. H. *et al.* Boron nitride dots In-situ embedded in a B_2O_3 matrix with the long lifetime Room-Temperature phosphorescence in dry and wet states. *Chem. Eng. J.* **417**, 129175 (2021).
209. Li, C. H., Zhu, J. K. & Wang, Q. C. Amorphous pure organic phosphorescent host-guest complexes with ultralong phosphorescence lifetime and high-temperature tolerance. *Dyes Pigm.* **204**, 110368 (2022).
210. Zhang, T., Wu, Y. A. & Ma, X. Tunable multicolor room-temperature phosphorescence including white-light

emission from amorphous copolymers. *Chem. Eng. J.* **412**, 128689 (2021).
211. Sun, J. *et al.* Combining π-conjugated groups by flexible alkyl chains for ultralong organic phosphorescence by photo-activation. *J. Lumin.* **247**, 118894 (2022).
212. Zhou, X. L. *et al.* 5,5-Dioxoyphenothiazine-based D-A-D type AIE molecules enabling persistent room temperature phosphorescence, white light emission and dual-mode mechanochromism. *Dyes Pigm.* **188**, 109193 (2021).
213. Gong, Y. Y. *et al.* Achieving persistent room temperature phosphorescence and remarkable mechanochromism from pure organic luminogens. *Adv. Mater.* **27**, 6195-6201 (2015).
214. Chong, K. C. *et al.* Structurally resemblant dopants enhance organic room-temperature phosphorescence. *Adv. Mater.* **34**, 2201569 (2022).
215. Yao, X. Y. *et al.* Room-temperature phosphorescence enabled through nacre-mimetic nanocomposite design. *Adv. Mater.* **33**, 2005973 (2021).
216. Louis, M. *et al.* Blue-light-absorbing thin films showing ultralong room-temperature phosphorescence. *Adv. Mater.* **31**, 1807887 (2019).
217. He, Z. H. *et al.* Achieving persistent, efficient, and robust room-temperature phosphorescence from pure organics for versatile applications. *Adv. Mater.* **31**, 1807222 (2019).
218. Xie, Y. J. *et al.* How the molecular packing affects the room temperature phosphorescence in pure organic compounds: ingenious molecular design, detailed crystal analysis, and rational theoretical calculations. *Adv. Mater.* **29**, 1606829 (2017).
219. Thomas, H. *et al.* Aromatic phosphonates: a novel group of emitters showing blue ultralong room temperature phosphorescence. *Adv. Mater.* **32**, 2000880 (2020).
220. Wang, C. *et al.* Poly(arylene piperidine) quaternary ammonium salts promoting stable long-lived room-temperature phosphorescence in aqueous environment. *Adv. Mater.* **34**, 2204415 (2022).
221. Dai, X. Y., Huo, M., Dong, X. Y., Hu, Y. Y. & Liu, Y. Noncovalent polymerization-activated ultrastrong near-infrared room-temperature phosphorescence energy transfer assembly in aqueous solution. *Adv. Mater.* **34**, 2203534(2022).
222. Dou, X. Y. *et al.* Color-tunable, excitation-dependent, and time-dependent afterglows from pure organic amorphous polymers. *Adv. Mater.* **32**, 2004768 (2020).
223. Fan, Y. Y. *et al.* Mobile phone flashlight-excited red afterglow bioimaging. *Adv. Mater.* **34**, 2201280(2022).
224. Wang, Y. *et al.* High performance of simple organic phosphorescence host–guest materials and their application in time-resolved bioimaging. *Adv. Mater.* **33**, 2007811 (2021).
225. Wang, T. *et al.* Aggregation-induced dual-phosphorescence from organic molecules for nondoped light-emitting diodes. *Adv. Mater.* **31**, 1904273 (2019).
226. Qian, C. *et al.* More than carbazole derivatives activate room temperature ultralong organic phosphorescence of benzoindole derivatives. *Adv. Mater.* **34**, 2200544 (2022).
227. Yang, Y. *et al.* Tunable photoresponsive behaviors based on triphenylamine derivatives: the pivotal role of π-conjugated structure and corresponding application. *Adv. Mater.* **33**, 2104002 (2021).
228. Cai, S. Z. *et al.* Visible-Light-Excited Ultralong Organic Phosphorescence by Manipulating Intermolecular Interactions. *Adv. Mater.* **29**, 1701244 (2017).
229. Zhang, J. Y. *et al.* Highly efficient and robust full-color organic afterglow through 2D superlattices embedment. *Adv. Mater.* **34**, 2206712 (2022).
230. Zhen, X. *et al.* Ultralong phosphorescence of water-soluble organic nanoparticles for in vivo afterglow imaging. *Adv. Mater.* **29**, 1606665(2017).
231. Ma, X. K. *et al.* Supramolecular pins with ultralong efficient phosphorescence. *Adv. Mater.* **33**, 2007476 (2021).

232. Yang, Y. J. *et al.* Tunable photoresponsive behaviors based on triphenylamine derivatives: the pivotal role of π-conjugated structure and corresponding application. *Adv. Mater.* **33**, 2104002 (2021).
233. Bhattacharjee, I. & Hirata, S. Highly efficient persistent room-temperature phosphorescence from heavy atom-free molecules triggered by hidden long phosphorescent antenna. *Adv. Mater.* **32**, 2001348 (2020).
234. Dang, Q. X. *et al.* Room-temperature phosphorescence resonance energy transfer for construction of near-infrared afterglow imaging agents. *Adv. Mater.* **32**, *2006752* (2020).
235. Tao, Y. *et al.* Resonance-activated spin-flipping for efficient organic ultralong room-temperature phosphorescence. *Adv. Mater.* **30**, *1803856* (2018).
236. Liu, H. *et al.* Multicolor-tunable room-temperature afterglow and circularly polarized luminescence in chirality-induced coordination assemblies. *Chem. Sci.* **13**, 13922-13929 (2022).
237. Zhao, Y. H., Ding, B. B., Huang, Z. Z. & Ma, X. Highly efficient organic long persistent luminescence based on host-guest doping systems. *Chem. Sci.* **13**, 8412-8416 (2022).
238. Jena, S. *et al.* Crystallization induced room-temperature phosphorescence and chiral photoluminescence properties of phosphoramides. *Chem. Sci.* **13**, 5893-5901 (2022).
239. Liu, Y. F. *et al.* Photo-induced ultralong phosphorescence of carbon dots for thermally sensitive dynamic patterning. *Chem. Sci.* **12**, 8199-8206 (2021).
240. Zhou, J. W. *et al.* Organic room-temperature phosphorescence from halogen-bonded organic frameworks: hidden electronic effects in rigidified chromophores. *Chem. Sci.* **12**, 767-773 (2021).
241. Mao, Z. *et al.* The methylation effect in prolonging the pure organic room temperature phosphorescence lifetime. *Chem. Sci.* **10**, 179-184 (2019).
242. Chai, Z. *et al.* Abnormal room temperature phosphorescence of purely organic boron-containing compounds: the relationship between the emissive behaviorand the molecular packing, and the potential related applications. *Chem. Sci.* **8**, 8336-8344 (2017).
